# Reconstructing the Timing and Dispersion Routes of HIV-1 Subtype B Epidemics in The Caribbean and Central America: A Phylogenetic Story

**DOI:** 10.1371/journal.pone.0069218

**Published:** 2013-07-09

**Authors:** Israel Pagán, África Holguín

**Affiliations:** 1 Centro de Biotecnología y Genómica de Plantas (UPM-INIA) and E.T.S.I. Agrónomos, Universidad Politécnica de Madrid, Madrid, Spain; 2 HIV-1 Molecular Epidemiology Laboratory, Microbiology Department, Hospital Ramón y Cajal-IRYCIS and CIBER-ESP, Madrid, Spain; Institut Pasteur, France

## Abstract

The Caribbean and Central America are among the regions with highest HIV-1B prevalence worldwide. Despite of this high virus burden, little is known about the timing and the migration patterns of HIV-1B in these regions. Migration is one of the major processes shaping the genetic structure of virus populations. Thus, reconstruction of epidemiological network may contribute to understand HIV-1B evolution and reduce virus prevalence. We have investigated the spatio-temporal dynamics of the HIV-1B epidemic in The Caribbean and Central America using 1,610 HIV-1B partial *pol* sequences from 13 Caribbean and 5 Central American countries. Timing of HIV-1B introduction and virus evolutionary rates, as well as the spatial genetic structure of the HIV-1B populations and the virus migration patterns were inferred. Results revealed that in The Caribbean and Central America most of the HIV-1B variability was generated since the 80 s. At odds with previous data suggesting that Haiti was the origin of the epidemic in The Caribbean, our reconstruction indicated that the virus could have been disseminated from Puerto Rico and Antigua. These two countries connected two distinguishable migration areas corresponding to the (mainly Spanish-colonized) Easter and (mainly British-colonized) Western islands, which indicates that virus migration patterns are determined by geographical barriers and by the movement of human populations among culturally related countries. Similar factors shaped the migration of HIV-1B in Central America. The HIV-1B population was significantly structured according to the country of origin, and the genetic diversity in each country was associated with the virus prevalence in both regions, which suggests that virus populations evolve mainly through genetic drift. Thus, our work contributes to the understanding of HIV-1B evolution and dispersion pattern in the Americas, and its relationship with the geography of the area and the movements of human populations.

## Introduction

At the end of 2011, 34 million people were estimated to be living with HIV worldwide, of which 2.5 million were new infections [1], [2]. For the last decade, The Caribbean has been reported to have the second highest regional HIV prevalence (1%), with 230.000 people living with AIDS. In spite of its geographical proximity, Central America has a considerably lower HIV prevalence (0.5%), with 1.4 million people infected by HIV [2]. Given these numbers, HIV remains a major health concern in Central America and The Caribbean.

Increasing effort has been devoted to gather information on the HIV epidemiology in The Caribbean and Central America. In these regions, the burden of HIV ranges from very low prevalence in Cuba (0.1%) or Mexico (0.3%) to a significant 2.3% in Belize or 3% in Bahamas, with prevalence of up to 5% in Jamaica and 7% in Santa Lucia for specific collectives [1]. This large variation in HIV prevalence might be explained by differences in social and geographical characteristics of human populations such as: (i) the studied populations, (ii) the main mode of virus transmission, and (iii) the access to antiretroviral therapy [2]. For instance, HIV prevalence in female sex workers varies from 2% in the Dominican Republic to 12% in some areas of Mexico [2]; and it has been estimated that more than one quarter of the 2 million injecting drug users in Latin America might be living with HIV [3]. In addition, the main mode of HIV transmission in The Caribbean is believed to be unprotected sex between men and women, but most HIV epidemics in Central America are associated with networks of men who have sex with men [4]. Exceptions to this rule are Bermuda and Puerto Rico where unsafe injecting drug use significantly contributes to the spread of HIV [2]. Finally, antiretroviral therapy (ARV) coverage largely varies depending on the country, reaching more than 80% of the eligible population in Cuba, but less than 40% in Panama [1], [2].

Disease epidemiology may influence the genetic structure of the virus populations [5], [6]. However, knowledge on the evolutionary history, the population dynamics and the genetic structure of HIV in Central America and The Caribbean is scarcer than in other parts of the world [7]. HIV-1 subtype B (HIV-1B) is the predominant variant in these regions [8]–[12], with the exception of Cuba [13], [14]. The few analyses of the genetic structure of the HIV-1B population in Central America, limited to individual countries, have not reported genetic differences with the HIV-1B populations of other geographical regions [8], [9], [15]. On the other hand, phylogenetic analyses of multinational sequences have revealed that Caribbean HIV-1B isolates are genetically distinct from the subtype B pandemic clade [12], [16], [17]. These results have been associated with geographical and social characteristics of the human population. For instance, it has been proposed that this genetic compartmentalization of the Caribbean population is the result of selective pressures for adaptation to heterosexual transmission mode on the basis of the lack of association between the HIV-1B phylogeny and the geographical origin of each sequence [12]. However, these analyses were performed using a limited number of sequences (n = 67) and countries (n = 4), and no quantitative approach was used.

Another underexplored aspect of the HIV-1B epidemic in Central America and The Caribbean, also linked to disease epidemiology, is the migration pattern of the virus [16], [17]. Migration is one of the major processes shaping the genetic structure of virus populations [18], and reconstructing dispersion routes provides fundamental understanding of the evolutionary dynamics of virus epidemics [19]. It is generally accepted that Haiti is the origin of the HIV-1B pandemic, which seems to be associated with human migration patterns [16], [17]. Despite the importance of The Caribbean in the HIV-1B epidemic, to date there has been no attempt to reconstruct the evolutionary history or the migration patterns of the virus in this region. It could be hypothesized that Haiti was also the origin of the epidemic within The Caribbean islands. However, Gilbert et al. [16] found evidence that most of the initial movements of HIV-1B out of Haiti resulted in dead-end infections, which raises the possibility of a different origin for The Caribbean epidemic. Moreover, the Haitian sequences seems not to fall in the basal positions of phylogenetic trees when only Caribbean sequences are considered [12]. Accordingly, some authors have argued that the introduction of HIV-1B in The Caribbean that caused the pandemic in the region might have come from North America [20], [21]. Thus, the origin and spread of the epidemic in the central region of the Americas is still unclear.

The objective of the current study was to investigate: i) the time scale of the HIV-1B evolution; ii) the genetic structure of HIV-1B populations; iii) the dispersion patterns that may have led to the current genetic structure of the HIV-1B populations, and its possible association with the geography and with human migrations in Central America and The Caribbean. To address these issues, we have combined Bayesian phylogenetic reconstruction techniques on a Coalescence Theory framework, with classical population genetics methods. These methodologies have been shown to yield valuable information about HIV-1 population-level processes, such as transmission networks [22], [23], or evolutionary dynamics in several geographic regions [24]–[26]. Using this approach, we present here an analysis of the genetic structure and a reconstruction of the evolutionary history of HIV-1 subtype B in Central America and The Caribbean, which is the first of this kind in these geographical regions. Our data provide insights on migration patterns and evolutionary potential of HIV-1B, which may help to understand the evolutionary forces shaping the HIV-1B epidemic in the studied regions, and worldwide.

## Materials and Methods

### Sequence and Prevalence Data

**Figure 1:**
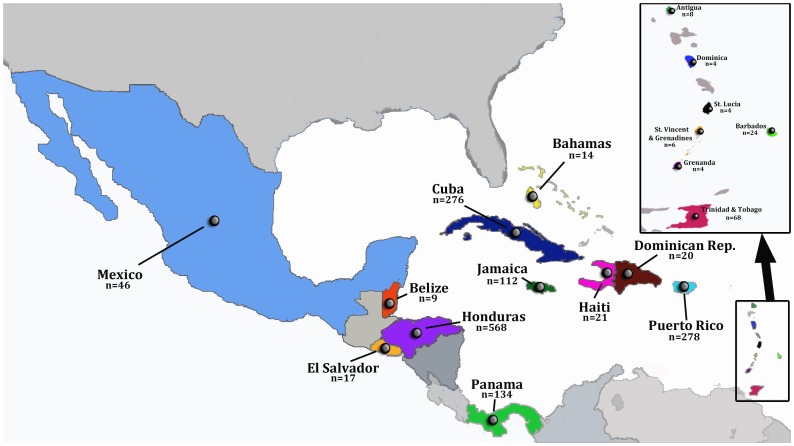
Geographic location of HIV-1B *pol_p_* sequences used in this study. Map shows the countries for which available sequences from HIV-1B infected patients were available (colored), and the number of sequences per country (n). Insert at the top right shows an expansion of the Eastern Caribbean islands. All sequences were sampled between 1996 and 2010.

All available HIV-1B sequences of individual patients from Central American and Caribbean countries, as well as the year of sampling of the virus isolate that led to each sequence, were recovered from Los Alamos National Lab HIV database [10]. No other HIV-1 subtypes or recombinant variants were considered in this study. Only countries with at least 4 available polymerase gene (*pol*) sequences were retained for analysis. Based on these criteria, a total of 1,610 HIV-1 partial *pol* sequences were selected. Sequences were collected from 774 HIV-1B infected individuals from five Central American countries between 2001 and 2010, and from 836 patients from thirteen Caribbean countries between 1996 and 2010. We focused our analysis on a region of the *pol* gene comprising the genomic region encoding for the viral protease (*PR)* and the fragment coding for the first 334 amino acid residues of the reverse transcriptase (*RT*) (1005 nucleotides, positions 2255–3259 of HXB2 subtype B reference strain, GenBank accession number K03455), from here on named *pol*_p_. The countries included in our analyses with the corresponding number of *pol_p_* sequences used are shown in Figure 1. A full list of GenBank accession numbers, countries of origin, and year of isolation, as well as the relevant publication associated with each sequence, is available as supplementary material (Table S1). Sequence alignments were constructed using MUSCLE 3.7 [27] and adjusted manually according to the amino acid sequences using Se-Al [28].

HIV-1B prevalence data in each country was obtained using previously reported data from 2001 to 2009, the period in which most of the sequences of our data sets were collected. The consulted sources reported highly similar prevalence values. Thus, for our analyses we used those reported by the United Nations [29], [30].

### Estimate of Nucleotide Substitution Rates and Divergence Time

Bioinformatics tools were applied to estimate nucleotide substitution rates and time to the most recent common ancestor (TMRCA) in our HIV-1 *pol*_p_ sequence data sets. The presence of temporal signal in the analyzed data sets is essential to the accurate estimate of these two parameters. Thus, we first assessed the strength of temporal signal in the Central American and in the Caribbean HIV-1 *pol*_p_ data sets. To do so, we constructed maximum-likelihood (ML) trees inferred with PhyML [31] using Subtree Pruning Regrafting (SPR) method, incorporating the best-fitted nucleotide substitution model as determined by jModelTest 0.1.1 [32]. Clock-like behavior of the data was then assessed by regressing root-to-tip distance in the ML tree against the date of sampling of each sequence using Path-O-Gen v1.3 [33]. This analysis effectively provides a measure of the amount of variation in genetic distances explained by sampling time. To confirm the temporal signal, the BEAST analyses described below were repeated on data sets in which sampling times were randomized so that temporal structure was disrupted. Runs for randomized data were repeated 10 times.

Rates of nucleotide substitutions per site per year (subs/site/year), and the TMRCA were estimated for both the Central American and the Caribbean data sets using the Bayesian Markov Chain Monte Carlo (MCMC) method available in the BEAST package v1.6.1 [34]. The best-fit model of nucleotide substitution in each case was determined using jModeltest. A relaxed (uncorrelated lognormal) molecular clock and the flexible Bayesian skyline model as a coalescent prior were also used. In all cases, the BEAST analyses were run until all relevant parameters converged, with 10% of the MCMC chains discarded as burn-in. Statistical confidence in the parameter estimates was represented by values for the 95% highest probability density (HPD) intervals around the marginal posterior parameter means. Substitution rates and TMRCAs were considered to be significantly different if the mean value of one estimate fell outside of the 95% HPD values of another (indicating that these rates/TMRCAs were drawn from different distributions). Maximum clade credibility (MCC) trees, with Bayesian posterior probability values providing a measure of statistical support at each node, were also inferred using BEAST. In these MCC trees, the date of the oldest node defining a cluster of sequences from a given country was selected as the TMRCA of that country.

### Analysis of Spatial Genetic Structure of the HIV-1B Populations

We also applied bioinformatics tools designed for comparative sequence analysis to explore the spatial genetic structure of the HIV-1B populations in Central America and The Caribbean. We followed three approaches:

1. We assessed the degree of association between country of origin and position of the sequence in the virus phylogenies using the Parsimony Score [PS, 35], the Association Index [AI, 36] and the Monophyletic Clade Size [MC, 37] statistics. PS and AI indicate the degree of phylogenetic clustering of sequences that come from the same country across the entire tree. MC assesses the association between country of origin and the virus phylogeny by estimating the size of the largest cluster of sequences coming from the same country. These analyses were performed using the program BaTS [37], which calculates empirical distributions of these statistics from the credible sample (posterior distribution) of trees provided by BEAST. Null distributions for the three statistics were generated by using 1000 data replications.
2. We estimated within- and between-countries genetic diversities, by calculating pairwise distances for each pair of countries, and using the Tamura-Nei nucleotide substitution model as implemented in MEGA 5 [38]. Standard errors of each genetic diversity measure were calculated based on 1000 replicate bootstrap. To determine if genetic diversity values were significantly different from each other, the 95% confidence interval (CI) was calculated in each case using the variance obtained by bootstrap [39] and assuming a normal distribution. We considered two genetic diversities as significantly different if the mean value of one estimate fell outside of the 95% CI of the other.

In addition, we calculated the percentage of the total genetic diversity explained by the between-countries genetic diversity using the *N_ST_* and the *F_ST_* coefficients. *N_ST_* was computed following the expression: 
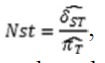
, where *δ_ST_* is the between-countries genetic diversity and*π_T_* the total genetic diversity [39]. *F_ST_* was calculated by Analysis of Molecular Variance (AMOVA), as implemented in Arlequin v.3.11 [40]. AMOVA was also utilized to test the differences between nucleotide diversities of the virus populations in each country. Significance of these differences was obtained by performing 1000 permutations.

1. We tested the hypothesis of isolation-by-distance [41], which predicts that the more geographically distant two populations of any organism are, the more genetically differentiated. We assessed the relationship between geographic and genetic distances using Mantel correlation tests as implemented in the isolation-by-distance (IBD) web service (http://ibdsw.sdsu.edu/~ibdws/) [42]. Matrices of geographical distances between pairs of countries were obtained by using GenAlEx6 [43], and matrices of genetic distances were obtained using MEGA 5 [38]. Geographic and genetic distances between pairs of populations were log transformed, and 1000 permutations were carried out to assess the significance of the correlations.

### Reconstruction of Spatio-temporal Dynamics

In order to know how HIV-1B has spread across Central American and Caribbean countries, the spatial dynamics of the virus populations through time were reconstructed for both regions using two different approaches:

1. We performed Population Graphs analyses [44] to represent relations among populations. Population Graphs use distances between individuals or populations to construct a network of nodes, which diameter represents the within-population genetic diversity, and edges linking the nodes that are proportional to population co-variances. The resulting network leaves populations with an independent structure unconnected. This method is nonhierarchical and allows for reticulate relationships.
2. We employed a model of discrete diffusion in a Bayesian inference framework as implemented in BEAST v1.6.1 [19]. This approach draws an explicit model describing how HIV-1B lineages, represented by the sampled sequences, migrated during their evolution across countries. The methodology employs continuous-time Markov chain models of discrete state evolution (meaning that rather than the individual GPS coordinates of each sequence being considered, all the sequences from the same country were assigned the same country state) to achieve ancestral reconstruction of these states. Inference of geographical locations throughout the evolutionary history along with a statistically meaningful measure of the confidence that can be associated with epidemiological linkage is achieved by using Bayes factors (BF) [45]. By connecting a stochastic model of discretized diffusion to standard models of sequence evolution, this method fully accounts for the uncertainty of every aspect in the underlying phylogeographic process [19]. Importantly, Population Graphs only take into account polymorphic sites, while Bayesian reconstructions use the whole sequence. The MCC trees for each data set were annotated with country state using the software TreeAnnotator (available in BEAST package). The tools available from http://beast.bio.ed.ac.uk/Google_Earth were used to produce a graphical representation of the spatio-temporal movement dynamics of ancestral HIV-1B sequences.

### Statistical Analysis

To discard that our substitution rate and TMRCA estimates could be biased due to either over- or under-representation of some countries, we performed BEAST analysis using a subset of sequences containing between 4 and 15 sequences per country (“balanced” subsets) in both the Central American and the Caribbean data sets. Thus, all sequences from countries with less than 15 *pol_p_* sequences were kept, and for countries with more than this number, fifteen sequences were randomly chosen. This randomization was repeated ten times and nucleotide substitution rates and TMRCAs were estimated in the ten “balanced” subsets.

We analyzed the correlation between the genetic diversity of the HIV-1B population in each country and the corresponding virus prevalence using Pearson’s correlation test. The presence of outliers, which potentially prevent significant linear correlation, was detected by calculating the studentized residual for each data point, dividing the residual by its standard deviation. Values outside the 95% CI of the Student t test distribution drawn with all of the studentized residuals were considered outliers. Statistical analyses were performed using SPSS 17.0 (SPSS Inc., Chicago, USA).

## Results

### Rapid Evolutionary Rates of the HIV-1B Populations in Central America and The Caribbean

Our analyses revealed significant root-to-tip correlations of sampling time *vs.* genetic distance for both the Central American (*r* = 0.42; *P* = 1×10^−5^) and the Caribbean (*r* = 0.30; *P* = 1×10^−5^) data sets (Figure S1). Therefore, the two *pol_p_* data sets contained sufficient temporal structure for reliable estimation of evolutionary rates and divergence times of the HIV-1B populations. This was confirmed by the significantly smaller mean and larger HPDs of the substitution rate estimates obtained for randomized data sets as compared with those from the real data (data not shown). Root-to-tip correlations considering the sequences of each country individually showed non-significant values in 4/5 and 10/13 countries in Central America and The Caribbean, respectively, plus no differences in rate estimates between real and randomized data sets. This prevented obtaining nucleotide substitution rates for the HIV-1B populations in most countries (see Table 1).

**Table 1:** Nucleotide substitution rate and TMRCA estimates for the *pol* fragment of 1,610 sequences obtained from HIV-1 infected patients from Centro-American and Caribbean countries.

	Country	n^a^	Date range	Substitution Rate^b^	MRCA^c^	*d* d	MC^e^	Prevalence (%)^f^	First HIV detection^g^
**Central America**	Honduras	568	2001–2010	4.02×10^−3^ (3.73–4.65×10^−3^)	1990 (1985–1996)	0.018±0.000	1×10^−3^	1.0	1985
	Panama	134	2004–2005	ND	1994 (1984–1998)	0.020±0.001	1×10^−3^	1.2	1984
	El Salvador	17	2010	ND	1996 (1990–1998)	0.031±0.001	1×10^−3^	0.8	1984
	Mexico	46	2001–2005	ND	1996 (1990–1998)	0.015±0.001	1×10^−3^	0.3	1983
	Belize	9	2004	ND	1998 (1990–2000)	0.020±0.001	1×10^−3^	2.3	1986
	***ALL***	***774***	***2001–2010***	***3.89×10^−3^ (2.89–4.72×10^−3^)***	***1990 (1985–1996)***	***0.022±0.004***		***0.5***	
**Caribbean**	Puerto Rico	276	1996–2005	2.80×10^−3^ (0.88–4.74×10^−3^)	1980 (1960–1986)	0.028±0.001	1×10^−3^	0.5*	1981
	Antigua	8	2000	ND	1980 (1960–1986)	0.023±0.002	7×10^−3^	0.7*	1985
	Cuba	276	1999–2010	3.64×10^−3^ (1.07–4.30×10^−3^)	1982 (1975–1985)	0.035±0.001	1×10^−3^	0.1	1986
	Jamaica	112	2001–2009	2.03×10^−3^ (0.87–4.23×10^−3^)	1983 (1981–1985)	0.030±0.001	1×10^−3^	1.8	1982
	St. Lucia	4	2000	ND	1983 (1981–1986)	0.017±0.001	1×10^−3^	0.1*	1985
	Bahamas	14	2004	ND	1984 (1982–1989)	0.045±0.002	1×10^−3^	3.1	1985
	Grenada	4	2000	ND	1984 (1981–1986)	0.001±0.000	3×10^−3^	-	1984
	Barbados	24	1996	ND	1984 (1982–1988)	0.022±0.001	1×10^−3^	1.0	1984
	Haiti	20	2004–2005	ND	1985 (1983–1987)	0.011±0.001	0.05	2.3	1983
	Dominican Republic	20	2002–2005	ND	1985 (1980–1990)	0.026±0.001	1×10^−3^	0.9	1983
	Trinidad & Tobago	68	2000–2003	ND	1985 (1983–1989)	0.027±0.001	1×10^−3^	1.4	1983
	St. Vincent &Grenadines	6	2000	ND	1985 (1983–1990)	0.023±0.003	3×10^−3^	0.9*	1984
	Dominica	4	2000	ND	1987 (1984–1990)	0.020±0.002	1×10^−3^	0.4*	1987
	***ALL***	***836***	***1996–2010***	***3.59×10^−3^ (3.03–4.24×10^−3^)***	***1980 (1960–1985)***	***0.035±0.004***		***1.0***	

a Number of partial *pol* sequences of each country.

b Nucleotide substitution rate (substitutions per site per year), with 95% HPD in parenthesis.

c Most Recent Common Ancestor (date) based on Figure 2 trees, with 95% HPD in parenthesis.

d Genetic distance (mean±standard error).

e Monophyletic clade size significance (*p*-value).

f Mean prevalence of HIV-1 infection between 2001–2010 (UNAIDS 2010). Asterisks indicate data extracted from the OECS HIV-AIDS report 2006.

g Year in which first case of AIDS was reported.

ND: Not-determined due to lack of temporal structure in the data set.

Estimated nucleotide substitution rates for the Central American and Caribbean data sets revealed similar mean values: 3.89×10^−3^ subs/site/year (95% HPD = 2.89×10^−3^ to 4.72×10^−3^) for the Central American population, and 3.59×10^−3^ subs/site/year (95% HPD = 3.03×10^−3^ to 4.24×10^−3^) for the Caribbean population (Table 1). The results from the “balanced” data sets did not differ from those obtained considering the complete data sets (Table S2). Hence, the HIV-1B populations in Central America and The Caribbean evolve rapidly, with no apparent effect of geographical location in their substitution rates.

### Age of HIV-1B Epidemic in The Caribbean and in Central America

TMRCA estimates indicated that the HIV-1B sampled diversity arose around 1990 in Central America (95% HPD = 1985–1996), and around 1980 in The Caribbean (95% HPD = 1960–1986). Therefore, our most conservative estimates indicate that the HIV-1B epidemic started not earlier than the 80 s in Central America, and not before 1960 in The Caribbean. Importantly, estimates of the “balanced” data sets yielded similar results (Table S2). Our results indicated that the HIV-1B epidemic in Central America seemed to have started earlier in Honduras (1990; 95% HPD = 1985–1996) than in Panama, El Salvador, and Mexico (1994–1996; 95% HPD = 1984–1998), with the most recent introduction occurring in Belize (1998; 95% HPD = 1990–2000). In The Caribbean, our TMRCA estimates indicated that the earliest epidemic occurred in the islands of Puerto Rico and Antigua (1980; 95% HPD = 1960–1986), and the latest in Dominica (1987; 95% HPD = 1984–1990), with the HIV-1B populations in the rest of the countries showing TMRCAs within this interval of seven years (Table 1). These values were not significantly different from those obtained for the countries for which TMRCAs could be estimated independently, thus validating our approach (not shown). Therefore, the HIV-1B epidemic seems to have started earlier in The Caribbean than in Central America, rapidly spreading between countries in both geographical regions (within a decade after the first introduction).

### Phylogenetic Clustering of HIV-1B Sequences in Central America and The Caribbean

Visual inspection of our MCC trees suggested a certain degree of genetic structure based on geographical location (Figure 2). Accordingly, both the AI and PS statistics revealed a significant association between the HIV-1B phylogeny and the country of origin of the sequences across the whole Central American and Caribbean phylogenies (*P*<1×10^−5^). The MC statistic also indicated a significant association for each country (*P*<0.05) (Table 1 and Figure 2). In addition, the three statistics showed a significant spatial genetic structure of the Caribbean population according to whether each sequence was originated in the Greater or Lesser Antilles (*P*<1×10^−3^). This indicated the existence of two major clusters: Cluster I, which grouped the majority of the sequences from the Lesser Antilles, Haiti plus half of the Jamaican sequences; and Cluster II, which included most of Greater Antilles sequences (The Bahamas, Cuba, Puerto Rico, Dominican Republic, and about half of the Jamaican sequences) (Figure 2B). MCC trees obtained for the “balanced” subsets of sequences presented similar distribution patterns (available upon request). Hence, HIV-1B isolates coming from the same geographical area tend to cluster together in the trees, indicating the existence of a spatial genetic structure in the HIV-1B populations analyzed.

**Figure 2:**
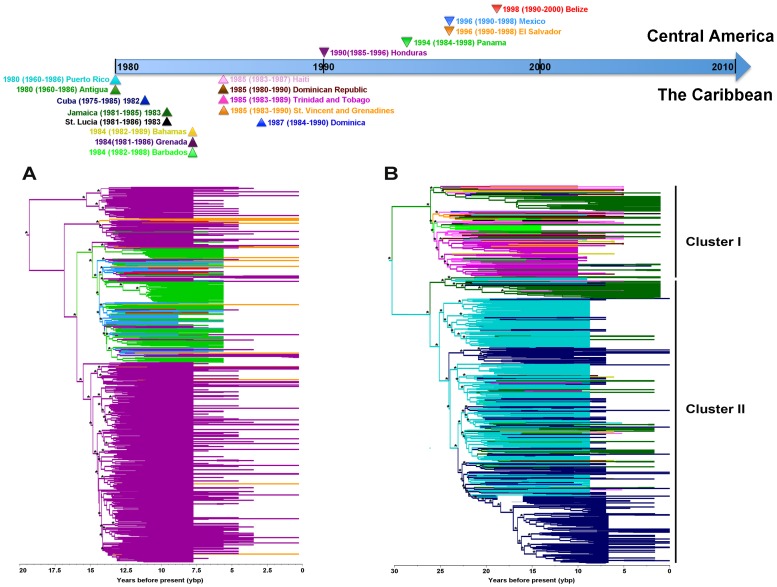
Maximum clade credibility phylogenies of the HIV-1B populations. Trees for the Central American (A) and The Caribbean (B) HIV-1 sequences are based on a fragment of the *pol* gene (1,005 nt). Branch tip times reflect the times of viral sampling. The trees are automatically rooted through the use of a relaxed molecular clock, and the total depth of the trees are the TMRCA of the HIV-1B Central American and Caribbean populations. Asterisks indicate nodes with posterior probabilities of >0.80. Branches are colored based on the country state of each node inferred using a discrete geographical model. Vertical back lines in panel B indicate the two mayor sequence clusters: I) Grouping sequences from the Eastern Caribbean islands, Haiti plus half of the Jamaican sequences. II) Grouping sequences from the Western Islands: Cuban, Puerto Rican and Dominican Republic, and half of the Jamaican and The Bahamas sequences.

### Genetic Differentiation of the Central American and Caribbean HIV-1B Populations According to the Country of Origin

Overall genetic diversities were generally very small in the HIV-1B populations analyzed (Table 1), being significantly smaller in the Central American than in the Caribbean population (0.022±0.004 *vs*. 0.035±0.004, respectively). Overall HIV-1B genetic diversities in each country were also generally smaller in the Central American than in the Caribbean region, varying in a 2-fold interval for the former (0.015±0.001 in Mexico to 0.031±0.001 in El Salvador), and in a 4-fold interval for the later (0.011±0.001 in Haiti to 0.045±0.002 in Bahamas). We excluded Grenada whose low diversity value seemed to be due to the limited number of sequences from this island (Table 1). These results indicate that the *pol_p_* region analyzed is subjected to strong selective constraints to genetic change, higher in the Central American than in the Caribbean population.

In addition, the mean within-country genetic diversity was generally lower than the mean between-countries diversity in both Central America (0.021±0.003 *vs*. 0.024±0.001; Table 2) and The Caribbean (0.024±0.003 *vs.* 0.030±0.002; Table 3). Analysis for each country showed a similar trend, with the exception of El Salvador in Central America (Table 2), and Bahamas, Jamaica and Cuba in The Caribbean (Table 3). The higher between-countries genetic diversity observed in the two HIV-1B populations under study is indicative of spatial genetic structure according to the country of origin. We validated these results by calculating *F_ST_* values using an AMOVA test, which showed a significant degree of spatial genetic structure in both The Caribbean (*F_ST_* = 0.10; *P* = 1×10^−5^) and Central America (*F_ST_* = 0.08; *P* = 1×10^−5^). Interestingly, while the mean within-country genetic diversity was similar in the Central American and the Caribbean data sets, mean HIV-1B between-country genetic diversity was higher in The Caribbean than in Central America. This is suggestive of a higher degree of spatial structure in the Caribbean area. Accordingly, *N_ST_* values revealed higher degree of spatial genetic structure based on the country of origin in The Caribbean than in Central America (0.28±0.11 *vs.* 0.16±0.09, respectively).

**Table 2:** Within- and between-country genetic distances for HIV-1 Centro-American populations.

	Belize	El Salvador	Honduras	Mexico	Panama	Total
**Belize**	0.020±0.001					
**El Salvador**	0.027±0.001	0.031±0.001				
**Honduras**	0.022±0.001	0.026±0.001	0.018±0.000			
**Mexico**	0.021±0.001	0.025±0.001	0.020±0.000	0.015±0.001		
**Panama**	0.024±0.001	0.029±0.001	0.023±0.000	0.022±0.000	0.020±0.001	
**Within**	0.020±0.001	0.031±0.001	0.018±0.000	0.015±0.001	0.020±0.001	0.021±0.003
**Between** a	0.024±0.001	0.027±0.001	0.023±0.001	0.022±0.001	0.025±0.002	0.024±0.001

Between-country genetic diversities were obtained by pairwise comparisons. Diagonal values represent within-country genetic diversity. Values are mean±standard deviation.

a Mean between-countries genetic diversity calculated as the mean of pairwise comparisons for each country.

**Table 3:** Within- and between-country genetic distances for HIV-1 Caribbean populations.

	Antigua	Bahamas	Dominica	St. Vincent	Grenada	St. Lucia	TrinidadTobago	Dominican Rep.	Haiti	Jamaica	Cuba	Barbados	Puerto Rico	Total
**Antigua**	0.023±0.002													
**Bahamas**	0.041±0.001	0.045±0.002												
**Dominica**	0.023±0.002	0.039±0.002	0.020±0.002											
**St. Vincent**	0.037±0.002	0.057±0.002	0.039±0.002	0.023±0.003										
**Grenada**	0.012±0.001	0.029±0.001	0.011±0.001	0.028±0.001	0.001±0.000									
**St. Lucia**	0.021±0.001	0.037±0.001	0.019±0.001	0.036±0.002	0.008±0.001	0.017±0.001								
**Trinidad Tobago**	0.027±0.001	0.044±0.001	0.026±0.001	0.040±0.002	0.015±0.001	0.023±0.001	0.027±0.001							
**Dominica Rep.**	0.025±0.001	0.042±0.001	0.024±0.001	0.041±0.001	0.013±0.001	0.022±0.001	0.028±0.001	0.026±0.001						
**Haiti**	0.019±0.001	0.035±0.001	0.017±0.001	0.031±0.002	0.006±0.001	0.015±0.001	0.020±0.001	0.020±0.001	0.011±0.001					
**Jamaica**	0.027±0.001	0.044±0.001	0.026±0.001	0.042±0.001	0.015±0.001	0.023±0.001	0.029±0.001	0.028±0.001	0.021±0.001	0.030±0.001				
**Cuba**	0.029±0.001	0.046±0.001	0.028±0.001	0.045±0.001	0.018±0.001	0.026±0.001	0.032±0.001	0.030±0.001	0.024±0.001	0.032±0.001	0.035±0.001			
**Barbados**	0.039±0.001	0.055±0.001	0.037±0.001	0.053±0.002	0.026±0.001	0.035±0.001	0.040±0.001	0.039±0.001	0.032±0.001	0.041±0.001	0.044±0.001	0.022±0.001		
**Puerto Rico**	0.031±0.001	0.048±0.001	0.030±0.001	0.046±0.001	0.019±0.001	0.027±0.001	0.033±0.001	0.032±0.001	0.025±0.001	0.034±0.001	0.036±0.000	0.045±0.001	0.028±0.001	
**Within**	0.023±0.002	0.045±0.002	0.020±0.002	0.023±0.003	0.001±0.000	0.017±0.001	0.027±0.001	0.026±0.001	0.011±0.001	0.030±0.001	0.035±0.001	0.022±0.001	0.028±0.001	0.024±0.003
**Between** a	0.028±0.002	0.043±0.002	0.027±0.003	0.041±0.002	0.017±0.002	0.027±0.001	0.030±0.002	0.029±0.002	0.020±0.003	0.030±0.003	0.031±0.003	0.041±0.002	0.034±0.003	0.030±0.002

Between-country genetic diversities were obtained by pairwise comparisons. Diagonal values represent within-country genetic diversity. Values are mean±standard deviation.

a Mean between-countries genetic diversity calculated as the mean of pairwise comparisons for each country.

Finally, Mantel tests showed no significant correlation between genetic and geographical distances among pairs of HIV-1B populations (countries) in neither of the two data sets analyzed (*r* = 0.36, *P* = 0.286 for Central America; *r* = 0.15, *P* = 0.806, for The Caribbean).

Taken together, these results provide evidence of spatial genetic structure in the Central American and the Caribbean HIV-1B populations. To further analyze which factors may shape the observed genetic structure, we analyzed the association between virus prevalence and genetic diversity, as well as the spatio-temporal dynamics of the HIV-1B populations in both regions.

### Association between HIV-1B within-country Genetic Diversity and Virus Prevalence in Central America and The Caribbean

**Figure 3:**
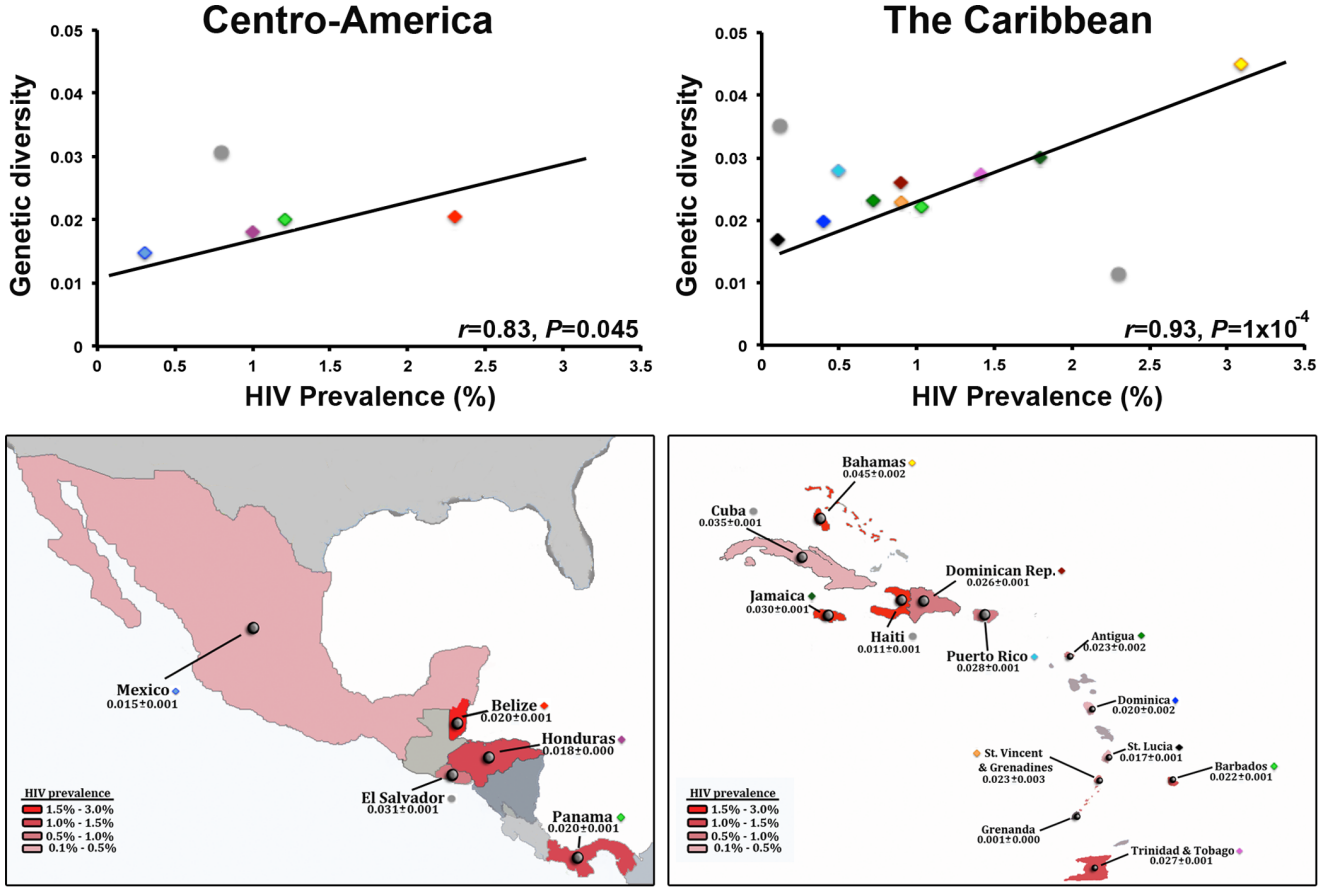
Correlation between within-country genetic diversity and prevalence of HIV-1 in Central America and The Caribbean. Each dot represents a country following the color codes in Figure 1. Grey dots indicate outlier countries eliminated from the analysis. Bottom maps show the genetic diversity of each country (mean±standard deviation), with countries colored based on the prevalence of HIV-1 infection.

For Central America, no significant correlation was found between HIV-1B within-country genetic diversity and prevalence (*r* = 0.05; *P* = 0.942) (Table 1 and Figure 3). However, this was solely due to the high genetic diversity of the virus population in El Salvador as compared with its prevalence, which was detected as an outlier of the distribution. When this country was eliminated from the analysis, a significant positive correlation was found between the two analyzed traits (*r* = 0.83; *P* = 0.045) (Figure 3). For The Caribbean, mean HIV-1B prevalence could be obtained for 12/13 countries. Using this data, no significant correlation was found between virus within-country genetic diversity and prevalence (*r* = 0.32; *P* = 0.304). Nevertheless, as for the Central American data set, this was due to outlier values in Cuba and Haiti. When these two countries were eliminated from the analysis, again a significantly positive correlation between HIV-1B genetic diversity and virus prevalence was found (*r* = 0.93; *P* = 1×10^−4^) (Figure 3).

### Routes and Migration Patterns of HIV-1B Populations in Central America and The Caribbean

**Figure 4:**
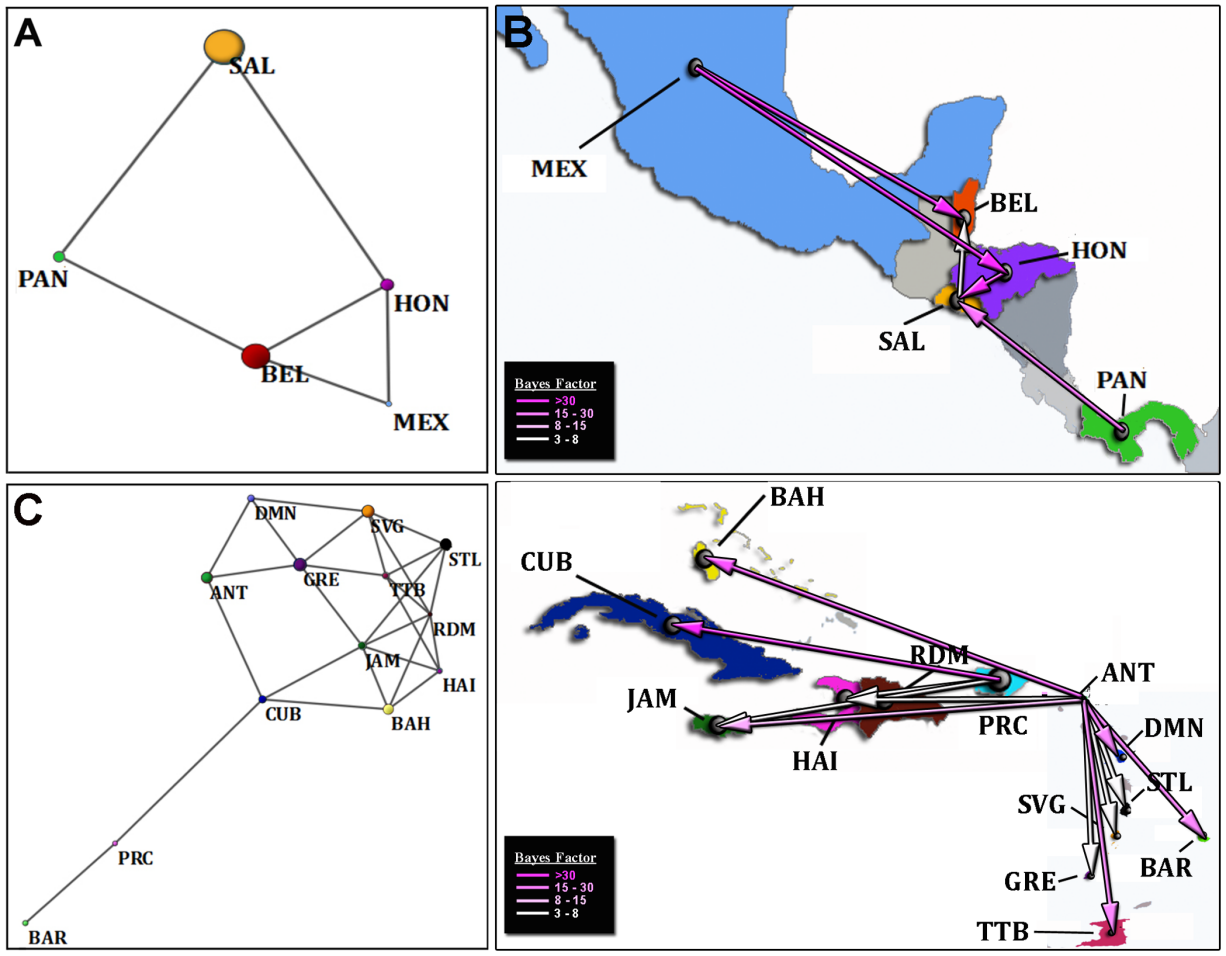
Genetic relations and representation of migration patterns of HIV-1B in Central America and The Caribbean. Genetic relations are represented with Population Graphs, where diameter of the nodes represents the within-country genetic diversity of the viral population. Edge length represents the among population genetic variation. No connectivity means no co-variation and therefore no genetic flow between countries. Representations of migration routes between HIV-1B populations are presented as virus movements implied by country state transitions along the branches of the MCC tree and are indicated using pink lines. Line colors indicate the overall Bayes Factor test support for epidemiological linkage between countries, and arrows indicate the direction of the movement. Google Earth is the original source of these representations. Original Google Earth images are accessible through Files S1 and S2. Each country is represented by a three-letter code. Central America (Panels A and B): Belize-BEL, El Salvador-SAL, Honduras-HON, Mexico-MEX, and Panama-PAN. The Caribbean (Panels C and D): Antigua-ANT, Bahamas-BAH, Barbados-BAR, Cuba-CUB, Dominica-DMN, Dominican Republic-RDM, Grenade-GRE, Haiti-HAI, Jamaica-JAM, Puerto Rico-PRC, St. Lucia-STL, St. Vincent and the Grenadines-SVG, Trinidad and Tobago-TTB.

The Population Graph constructed with the Central American sequences showed connections between the countries in the central region (Belize, Honduras and El Salvador), which were also connected with the Northern and Southern countries. No connection was found between Mexico and Panama, the two more distant countries (Figure 4A). Interestingly, the most important nodes in the network seemed to be Honduras and Belize as they presented the highest number of connections, which is suggestive of a stronger genetic flow in these countries. This was in agreement with the higher within-country genetic diversity (diameter of the nodes) in the central countries as compared with Mexico and Panama, and with our calculated values of within-country genetic diversity. HIV-1B populations from Honduras and Belize seemed to be closer to those from Mexico (shorter internode branches), which also suggests a more intense genetic flow between the central and Northern areas (Figure 4A). The Bayesian analysis was largely in accordance with Population Graphs, revealing that HIV-1B seemed to have initially appeared and spread within the central region (Honduras, El Salvador BF>7), with later and simultaneous migrations from Mexico and Panama (BF>17) to Honduras, El Salvador and Belize (BF>7), and from El Salvador to Belize (BF = 6.6). Again, there was strong evidence of genetic flow between Mexico, Honduras and Belize, but no link between Mexico and Panama (Figure 4B and File S1). Importantly, estimates of the “balanced” data sets yielded similar results, with 7/10 replicates finding Honduras, and 3/10 finding El Salvador, as the origin of the epidemic (Table S2). Together, these results indicate that in Central America HIV-1B was dispersed following a radial pattern.

For The Caribbean, Population Graphs showed a network formed by most of the countries with the exception of Barbados and Puerto Rico (Figure 4C). In agreement with our inferred phylogeny, the Lesser Antilles (Antigua, Dominica, Grenade, St. Lucia, St. Vincent, and Trinidad & Tobago), and the Greater Antilles (Bahamas, Cuba, Dominican Republic, Haiti, Jamaica, Puerto Rico) were significantly disconnected based on a binomial test, forming two subgraphs (*P*<0.050 and *P*<0.033, respectively). This indicates a higher genetic flow among closest countries than among those separated by larger distances, and a clear East-West division. Despite Barbados can be counted as part of the Lesser Antilles, it seemed to be genetically disconnected from any other country with the only exception of its association with Puerto Rico (Figure 4C). The Bayesian reconstruction also indicated clearly distinguishable migration areas within the Greater and within the Lesser Antilles (Figure 4D and File S2). HIV-1B introduction in these two areas seemed to have occurred through Puerto Rico, with an early jump to Antigua (BF = 11.5) (Table 1 and Figure 2). Based on our analysis, the spread of the virus could have occurred in two waves. During the early 80 s the virus would have jumped from Puerto Rico to Cuba and Haiti (BF>128), and from Antigua to Bahamas, Jamaica and Haiti (BF between 4 and 25). In the same period, a secondary dispersion event in the Greater Antilles would have occurred between Haiti and Dominican Republic (BF = 173). Meanwhile, in the Lesser Antilles the virus would have jumped from Antigua to Trinidad and Tobago, Barbados and St. Lucia (BF between 4 and 12). In a second wave during the late 80 s, the virus would have spread from Puerto Rico to Jamaica in the Greater Antilles (BF = 4.5), and from Antigua to St. Vincent, Dominica and Grenada in the Western Lesser Antilles (BF>4). It is worth noting that at odds with the Population Graphs, Barbados was included within the migration area of the Lesser Antilles, with detectable migration events only with Antigua (Figure 2, Figure 4D and File S2). Again, estimates of the “balanced” data sets yielded similar results, and 8/10 replicates found Puerto Rico as the origin of the epidemic (Table S2). Thus, HIV-1B spread in the Caribbean islands seemed to have occurred mostly through short-distance movements in two clearly distinguishable areas: one in the Lesser Antilles following a North-South direction, and another in the Greater Antilles following East-West diffusion.

## Discussion

In this work we provide an analysis of the genetic structure and the spatio-temporal dynamics of the HIV-1 subtype B populations in Central America and The Caribbean. Both HIV-1B populations seems to be characterized by a recent origin, rapid evolutionary dynamics, and are genetically structured according to the country of origin. This genetic structure appears to be the result of the virus dispersion pattern through short-distance movements, and of a limited genetic flow between countries due to geographical barriers and to preferential movement of human populations among culturally related countries.

Our results are derived from both phylogenetic inferences based on Coalescence Theory and from classical population genetics analyses. By combining these two partially overlapping approaches, we have been able to obtain complementary information, as well as to validate our results, which make our conclusions more robust. In addition, the utilized data set (the largest at the time of this study) included *pol* sequences from at least two thirds of the countries in each of the two analyzed geographical regions. Although the *pol_p_* fragment is known to contain amino acid positions associated with ARV resistance, which would potentially bias our conclusions, the presence of drug resistance mutations has been reported not to affect HIV-1 phylogenetic reconstructions [22, 46. Finally, although samples were collected along a relatively short time span (10 to 14 years), this is in the same range than similar studies in other HIV-1 populations [25], [47], [48]. Moreover, by limiting our study to HIV-1 subtype B, the prevalent HIV-1 variant in these countries; we have improved the accuracy of our estimates of evolutionary parameters [49].

### High Evolutionary Rates and Low Genetic Diversity in the *pol* Gene

Estimates of evolutionary rates indicated rapid evolutionary dynamics of the HIV-1B populations analyzed. These estimates may be overestimates due to the short time span considered, and to the presence of deleterious mutations still not purged by selection, which seem to be a common component in intra-specific analyses of evolutionary rates [50]. However, the inferred substitution rates are within the range of those reported for other HIV-1 populations around the world using equivalent fragment of the *pol* gene [25], [51], [52], [53]. The observed high evolutionary rates were accompanied by a low genetic diversity in the *pol_p_* fragment (Table 1), indicating that this fragment is subjected to strong selective constraints. This is not surprising since the *RT* and the *PR* proteins are involved in vital functions for the virus life cycle such as replication or infectivity [54]. In agreement with our results, other studies have also found that the *pol* gene is highly conserved [e.g. 55–57]. It is not obvious how high evolutionary rates and low genetic diversity in the *pol_p_* fragment coexist. It could be hypothesized that this might be the result of multiple mutations occurring in a reduced number of polymorphic sites [39]. Thus, even with fast accumulation of mutations over time, nucleotide changes between pairs of sequences would be located in just a few sites, which would result in low pairwise genetic diversity. Indeed, the *RT* and the *PR* proteins require full preservation of at least two thirds of their sequence in order to remain functional [55], [56].

### The Caribbean and Central American HIV-1B Populations are Genetically Structured According to the Country of Origin

Phylogenetic trees and AMOVA analyses indicated that the studied HIV-1B populations were genetically structured according to the geographical location (country). Genetic bottlenecks during transmission would generate the observed phylogenetic clustering by two mechanisms: (i) Selective pressures exerted, for instance, by the host’s immune system and/or ARV therapies would be different in each country [57]. As a consequence, the more fit HIV-1B variants would differ between countries, generating the observed spatial structure. There are several arguments against this idea. First, there is a lack of evidence of such differential selection [12], [16]. Second, strong pressures selecting for variants with resistance to ARV drugs would have kept relatively constant the genetic diversity of the virus population regardless of its prevalence. That is, in spite of differences in the number of infected individuals between countries, only a few variants harboring drug resistance mutations would account for most of the HIV-1B infections in each country. However, we observed a positive correlation between genetic diversity and prevalence. (ii) Effect of genetic drift [57]. Bottlenecks during horizontal (individual to individual of the same generation) and vertical (mother to child) transmission are a well-known feature of the HIV pandemic [7], [16]. Random transmission of HIV-1B variants due to such bottlenecks during between-countries virus jump, and/or during within-country virus transmission would have generated the observed spatial genetic structure. Interestingly, the positive association found between virus prevalence and population genetic diversity, indicating that the higher the frequency of infected individuals the higher the number of variants in the population, supports the role of genetic drift in shaping the genetic structure of the HIV-1B population in Central America and The Caribbean.

Previous analyses of the Caribbean and the Central American HIV-1B populations have failed in finding sequence clustering according to the country of origin [12], [16], [17], which is probably due to the limited sample size used, both in terms of the number of countries and of the number of sequences. Based on this result, it has been proposed that the evolution of the Caribbean HIV-1B population is driven by adaptation to the main transmission mode rather than to genetic drift due to transmission bottlenecks [12]. Accordingly, the HIV-1B population from Latin America and The Caribbean, which have different main transmission modes, seems to be genetically different [17]. Although we found evidence of genetic structure at the country level in the Caribbean HIV-1B population, this is not incompatible with previous results. Rather, selection for transmission mode and transmission bottlenecks might be shaping the genetic structure of the virus population at two different levels. Adaptation to transmission mode would act at a larger inter-regional scale (i.e. geographical region scale), causing the genetic differentiation of The Caribbean population from the HIV-1B pandemic clade. Transmission bottlenecks would act at a smaller intra-regional scale (i.e. country scale), causing the genetic differentiation of the HIV-1B population between countries. Remarkably, our between-countries *F_ST_* values and those between Latin American and Caribbean HIV-1B populations are in the same range, suggesting that the strength of both forces in generating the genetic spatial structure at the two spatial scales could be similar.

It could be argued that our study does not take into account the human collective from which sequences were obtained (i.e. infants or adults; female sex workers or men who have sex with men, etc.). Thus, the observed spatial structure might be just the consequence of having different collectives sampled in different countries. However, in the majority of the countries there were representatives of two or more of these collectives. Moreover, sequences within a given country did not generally group according to the human collective sampled (not shown). Thus, it is unlikely that the spatial genetic structure in the analyzed regions is an artifact.

### HIV-1B Dispersion Pattern in Central America

In Central America, both the Population Graph and the Bayesian phylogeographic reconstruction indicated that HIV-1B seems to have arisen in the central area (Honduras), and spread to the neighboring countries (El Salvador, Belize) through short-distance movements. The epidemiological link between Honduras and El Salvador is not surprising, since population movements between these two countries has been constant since the early 20^th^ century [58]. During the 80 s, Salvadorans and Hondurans emigrated northwards in great numbers. Although the destination of most of them was Mexico and the US, Belize accepted many migrants, especially from El Salvador [59], which would explain the link between the HIV-1B populations of these two countries (Figure 4B). Interestingly, Honduras, El Salvador and Belize seem to have had secondary introductions both from North (Mexico) and South (Panama) through long-distance radial movements. This may reflect introductions from other geographical areas such as North America and South America, perhaps associated with emigrants returning to their countries. Reported evidence of the link between the HIV-1B epidemics in the US and Mexico [60]–[62], or between epidemics in Panama and South America [8], would be compatible with this idea. Importantly, considering the relative long period of asymptomatic infection of HIV-1B, we should expect that the first AIDS reports must occurred around 8–10 years later of the true TMRCAs. However, the year in which the first HIV-1 case was reported in Honduras and Panama is in the lower bound of the HPD interval of our estimated TMRCAs [63] (Table 1), and for El Salvador, Belize and Mexico, TMRCA estimates are slightly more recent than the first case reported [63] (Table 1). Thus, we might be consistently underestimating TMRCA values. This does not necessarily affect the observed migration patterns, as the error is similar in all countries. TMRCA underestimation is probably due to the short time span of our data set rather than to a lack of sequence representativeness. Although we cannot discard that the small number of sequences in some countries does not reflect the genetic diversity of the virus population, in most countries the calculated genetic diversity matches what would be expected based on the virus prevalence, suggesting that limited sample size might not have a great effect in our reconstructions. Finally, the observed radial migration pattern might also explain the absence of correlation between geographic and genetic distances in our data set. The epidemic linkage between distant locations may have disrupted this correlation.

### HIV-1B Dispersion Pattern in The Caribbean

In The Caribbean, the Population Graph and the Bayesian reconstruction detected two clearly distinguishable migration areas, with little genetic exchange between them: one comprising the Greater Antilles, and another including the Lesser Antilles. This indicates that the dispersion of HIV-1B mainly occurred through short-distance movements. Thus, geographic barriers to virus movements seem to have played an important role in the dispersion of the HIV-1B epidemic in The Caribbean.

Our phylogeographic reconstruction indicated that the HIV-1B epidemic in The Caribbean could have been initially originated in Puerto Rico and Antigua, further spreading West and South. The epidemiological link between these two islands is not unexpected due to their geographical proximity, which makes Puerto Rico one of the major destinations for Antiguan migrants. Puerto Rico and the US have a shared HIV-1B epidemic [64], and it is believed that the US was the origin of the Puerto Rican HIV-1B epidemic [65]. However, there is no clear evidence supporting this idea. To date, it is thought that the HIV-1B pandemic was originated in Haiti in the 1960 s, when Haitian infected workers returned from Africa [66]. Phylogenetic analyses based on the *gag* and *env* genes have supported this idea [16]. Consequently, it could be assumed that Haiti is the origin of the HIV-1B epidemic in The Caribbean. However, other reposts have argued on a more recent introduction during the 80 s as a consequence of the sexual tourism from the US [20], [21]; and reconstructions of Caribbean HIV-1B phylogenies based on the *pol* gene have indicated that the Haitian sequences are not in the deepest branches of the tree [12]. Our results are compatible with this second hypothesis, but do not reject an initial introduction of HIV-1B in Haiti from Africa. It might be possible that the introduction in Haiti in the 60 s resulted in dead-end infections in The Caribbean islands, but succeeded jumping to North America as suggested by Gilbert et al. [16]. Later, a second introduction from the US through Puerto Rico during the 80 s, this time successful, would have generated the epidemic in The Caribbean. Unfortunately, with the existing data it is not possible to test this hypothesis, as *pol* sequences of the oldest Haitian isolates are not available, and could not be considered in our work. Analysis of HIV-1B dispersion patterns using a data set including older *pol* Haitian and US sequences would allow a fine time-scaled reconstructions of the epidemiological links between the US and The Caribbean, which would contribute to reconcile previous results and ours.

The reconstruction presented here has additional limitations: the number of sequences from each country is highly variable. The small sample size in some countries might not be representative of the virus population, while large sequence numbers in other countries may overweight their importance in the evolutionary history of HIV-1B. This may impose a strong bias in our TMRCA estimates and in the inferred migration routes. It is possible that we could not reveal some epidemiological links and/or overrepresented others. Indeed, in some countries the TMRCA values are more recent than the first case reported. Although we cannot rule out these factors as potential confounders, three lines of evidence support at least in part our reconstruction. First, although most of the genetic diversity of the HIV-1B population seems to have been generated since the 80 s, our most conservative TMRCA estimates trace the origin of the epidemic to the 60 s, in agreement with previous results [16]. Second, reconstructions using the “balanced” data sets, which contain a similar number of sequences per country, are largely in agreement with results of the “unbalance” data set. Third, the reported date of the first HIV case in each country falls within the HPD interval of our divergence time estimates, and in many cases our most conservative estimates (lower bound of the HPD) range between 3 and 10 years earlier than the first reported case, in accordance with the HIV asymptomatic period. Thus, our results generally match with the known epidemiological history of the virus.

Regardless of the origin of the HIV-1B epidemic, it is worth noting that we found an epidemiological link between Puerto Rico and all the other Western islands that are considered Latin American countries (Cuba, Haiti and Dominican Republic). Similarly, the Antigua HIV-1B population was linked to all the Eastern Caribbean islands, which were primarily ‘British-colonized’. This may also explain the association of the Antiguan with the Jamaican and Bahamas virus populations. Therefore, the inferred HIV-1B migration patterns indicate that, besides geography, cultural characteristics and migration patterns of the human population are also relevant determinants of the virus epidemiology and evolution in The Caribbean.

## Supporting Information

Figure S1 **Root-to-tip regressions of the Central American (A) and the Caribbean (B) HIV-1B data sets.** Regression of root-to-tip distance (inferred from ML trees) against year of isolation were calculated using the HIV-1B *pol_p_* data sets. The correlation coefficient and the significance of this correlation are shown in each panel.
(TIF)

Table S1 **Summary of sequence data, country of origin and date of isolation of HIV-1B isolates.**
(DOC)

Table S2 **Nucleotide substitution rate, TMRCA and HIV-1B epidemic geographical origin estimates for the ten “balanced” replicates of the** ***pol_p_*** **data sets from Central American (n = 69), and Caribbean (n = 155) countries.**
(DOCX)

File S1 **Migration patterns of HIV-1B in Central America.** Google Earth graphical representation of HIV-1B migration patterns obtained using BEAST v1.6.1. Representations of the strength of the epidemiological linkage and country codes as in Figure 4.
(KML)

File S2 **Migration patterns of HIV-1B in The Caribbean.** Google Earth graphical representation of HIV-1B migration patterns obtained using BEAST v1.6.1. Representations of the strength of the epidemiological linkage and country codes as in Figure 4.
(KML)
