# Supplementary figures and images for: Reconstructing the Timing and Dispersion Routes of HIV-1 Subtype B Epidemics in The Caribbean and Central America: A Phylogenetic Story
Source: PLoS One. 2013 Jul 9;8(7):e69218. doi: 10.1371/journal.pone.0069218 (PMC3706403; doi:10.1371/journal.pone.0069218)

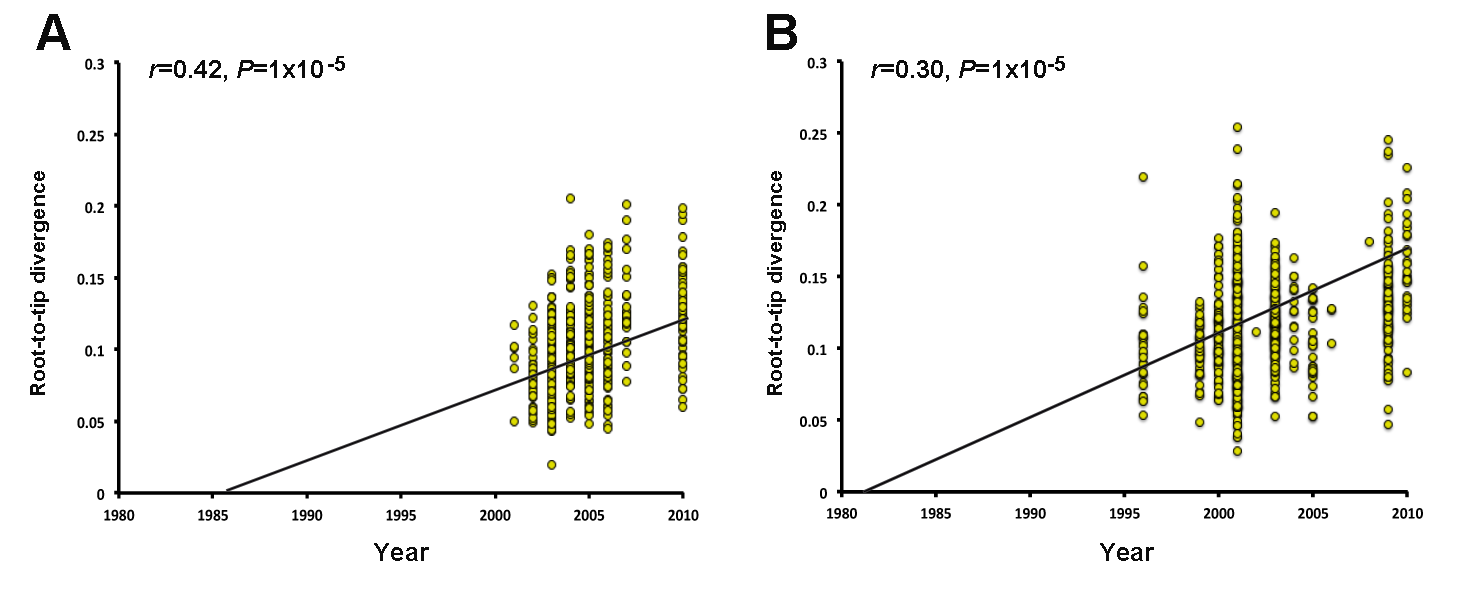

Supplement: Figure S1 — Root-to-tip regressions of the Central American (A) and the Caribbean (B) HIV-1B data sets. Regression of root-to-tip distance (inferred from ML trees) against year of isolation were calculated using the HIV-1B polp data sets. The correlation coefficient and the significance of this correlation are shown in each panel. (TIF) [file pone.0069218.s001.tif]
