# Supplementary material for: Reconstructing the Timing and Dispersion Routes of HIV-1 Subtype B Epidemics in The Caribbean and Central America: A Phylogenetic Story
Source: PLoS One. 2013 Jul 9;8(7):e69218. doi: 10.1371/journal.pone.0069218 (PMC3706403; doi:10.1371/journal.pone.0069218)
Supplement: Table S1 — Summary of sequence data, country of origin and date of isolation of HIV-1B isolates. (DOC) [file pone.0069218.s002.doc]

**Table S1.** Summary of sequence data, country of origin and date of isolation of HIV-1B isolates.

| **Accesion Nº** | **Isolate origin** | **Date** | **Reference** |
| --- | --- | --- | --- |
|  |  |  |  |
| **Central America** | |  |  |
| HQ599237 | Belize | 2004 | Parham et al. 2011 |
| HQ599238 | Belize | 2004 | Parham et al. 2011 |
| HQ599239 | Belize | 2004 | Parham et al. 2011 |
| HQ843022 | Belize | 2004 | Parham et al. 2011 |
| HQ843024 | Belize | 2004 | Parham et al. 2011 |
| HQ843025 | Belize | 2004 | Parham et al. 2011 |
| HQ843026 | Belize | 2004 | Parham et al. 2011 |
| HQ843028 | Belize | 2004 | Parham et al. 2011 |
| HQ843029 | Belize | 2004 | Parham et al. 2011 |
|  |  |  |  |
| HQ010650 | El Salvador | 2010 | Holguín et al. 2011 |
| HQ010651 | El Salvador | 2010 | Holguín et al. 2011 |
| HQ010653 | El Salvador | 2010 | Holguín et al. 2011 |
| HQ010654 | El Salvador | 2010 | Holguín et al. 2011 |
| HQ010655 | El Salvador | 2010 | Holguín et al. 2011 |
| HQ010657 | El Salvador | 2010 | Holguín et al. 2011 |
| HQ010658 | El Salvador | 2010 | Holguín et al. 2011 |
| HQ010659 | El Salvador | 2010 | Holguín et al. 2011 |
| HQ010660 | El Salvador | 2010 | Holguín et al. 2011 |
| HQ010661 | El Salvador | 2010 | Holguín et al. 2011 |
| HQ010662 | El Salvador | 2010 | Holguín et al. 2011 |
| HQ010663 | El Salvador | 2010 | Holguín et al. 2011 |
| HQ010664 | El Salvador | 2010 | Holguín et al. 2011 |
| HQ010665 | El Salvador | 2010 | Holguín et al. 2011 |
| HQ010666 | El Salvador | 2010 | Holguín et al. 2011 |
| HQ010671 | El Salvador | 2010 | Holguín et al. 2011 |
| HQ010672 | El Salvador | 2010 | Holguín et al. 2011 |
|  |  |  |  |
| DQ364457 | Honduras | 2004 | Castro et al. 2005 |
| EU312637 | Honduras | 2003 | Lloyd et al. 2008 |
| EU312638 | Honduras | 2003 | Lloyd et al. 2008 |
| EU312639 | Honduras | 2003 | Lloyd et al. 2008 |
| EU312640 | Honduras | 2003 | Lloyd et al. 2008 |
| EU312641 | Honduras | 2003 | Lloyd et al. 2008 |
| EU312642 | Honduras | 2003 | Lloyd et al. 2008 |
| EU312643 | Honduras | 2003 | Lloyd et al. 2008 |
| EU312644 | Honduras | 2003 | Lloyd et al. 2008 |
| EU312645 | Honduras | 2003 | Lloyd et al. 2008 |
| EU312646 | Honduras | 2003 | Lloyd et al. 2008 |
| EU312647 | Honduras | 2003 | Lloyd et al. 2008 |
| EU312648 | Honduras | 2003 | Lloyd et al. 2008 |
| EU312649 | Honduras | 2003 | Lloyd et al. 2008 |
| EU312650 | Honduras | 2003 | Lloyd et al. 2008 |
| EU312651 | Honduras | 2003 | Lloyd et al. 2008 |
| EU312652 | Honduras | 2003 | Lloyd et al. 2008 |
| EU312653 | Honduras | 2003 | Lloyd et al. 2008 |
| EU312654 | Honduras | 2003 | Lloyd et al. 2008 |
| EU312655 | Honduras | 2003 | Lloyd et al. 2008 |
| EU312656 | Honduras | 2003 | Lloyd et al. 2008 |
| EU312657 | Honduras | 2003 | Lloyd et al. 2008 |
| EU312658 | Honduras | 2003 | Lloyd et al. 2008 |
| EU312659 | Honduras | 2003 | Lloyd et al. 2008 |
| EU312660 | Honduras | 2003 | Lloyd et al. 2008 |
| EU312661 | Honduras | 2003 | Lloyd et al. 2008 |
| EU312662 | Honduras | 2003 | Lloyd et al. 2008 |
| EU312663 | Honduras | 2003 | Lloyd et al. 2008 |
| EU312664 | Honduras | 2003 | Lloyd et al. 2008 |
| EU312665 | Honduras | 2003 | Lloyd et al. 2008 |
| EU312666 | Honduras | 2003 | Lloyd et al. 2008 |
| EU312667 | Honduras | 2003 | Lloyd et al. 2008 |
| EU312669 | Honduras | 2003 | Lloyd et al. 2008 |
| EU312670 | Honduras | 2003 | Lloyd et al. 2008 |
| EU312671 | Honduras | 2003 | Lloyd et al. 2008 |
| EU312672 | Honduras | 2003 | Lloyd et al. 2008 |
| EU312673 | Honduras | 2003 | Lloyd et al. 2008 |
| EU312674 | Honduras | 2003 | Lloyd et al. 2008 |
| EU312675 | Honduras | 2003 | Lloyd et al. 2008 |
| EU312676 | Honduras | 2003 | Lloyd et al. 2008 |
| EU312677 | Honduras | 2003 | Lloyd et al. 2008 |
| EU312678 | Honduras | 2003 | Lloyd et al. 2008 |
| EU312679 | Honduras | 2003 | Lloyd et al. 2008 |
| EU312680 | Honduras | 2003 | Lloyd et al. 2008 |
| EU312681 | Honduras | 2003 | Lloyd et al. 2008 |
| EU312682 | Honduras | 2003 | Lloyd et al. 2008 |
| EU312683 | Honduras | 2003 | Lloyd et al. 2008 |
| EU312684 | Honduras | 2003 | Lloyd et al. 2008 |
| EU312685 | Honduras | 2003 | Lloyd et al. 2008 |
| EU312686 | Honduras | 2003 | Lloyd et al. 2008 |
| EU312687 | Honduras | 2003 | Lloyd et al. 2008 |
| EU312688 | Honduras | 2003 | Lloyd et al. 2008 |
| EU312689 | Honduras | 2003 | Lloyd et al. 2008 |
| EU312690 | Honduras | 2003 | Lloyd et al. 2008 |
| EU312691 | Honduras | 2003 | Lloyd et al. 2008 |
| EU312692 | Honduras | 2003 | Lloyd et al. 2008 |
| EU312693 | Honduras | 2003 | Lloyd et al. 2008 |
| EU312694 | Honduras | 2003 | Lloyd et al. 2008 |
| EU312695 | Honduras | 2003 | Lloyd et al. 2008 |
| EU312696 | Honduras | 2003 | Lloyd et al. 2008 |
| EU312697 | Honduras | 2003 | Lloyd et al. 2008 |
| EU312698 | Honduras | 2003 | Lloyd et al. 2008 |
| EU312699 | Honduras | 2003 | Lloyd et al. 2008 |
| EU312700 | Honduras | 2003 | Lloyd et al. 2008 |
| EU312701 | Honduras | 2003 | Lloyd et al. 2008 |
| EU312703 | Honduras | 2003 | Lloyd et al. 2008 |
| EU312704 | Honduras | 2003 | Lloyd et al. 2008 |
| EU312705 | Honduras | 2003 | Lloyd et al. 2008 |
| EU312706 | Honduras | 2003 | Lloyd et al. 2008 |
| EU312707 | Honduras | 2003 | Lloyd et al. 2008 |
| EU312708 | Honduras | 2003 | Lloyd et al. 2008 |
| EU312710 | Honduras | 2003 | Lloyd et al. 2008 |
| EU312711 | Honduras | 2003 | Lloyd et al. 2008 |
| EU312712 | Honduras | 2003 | Lloyd et al. 2008 |
| EU312713 | Honduras | 2003 | Lloyd et al. 2008 |
| EU312714 | Honduras | 2003 | Lloyd et al. 2008 |
| EU312715 | Honduras | 2003 | Lloyd et al. 2008 |
| EU312716 | Honduras | 2003 | Lloyd et al. 2008 |
| EU312717 | Honduras | 2003 | Lloyd et al. 2008 |
| EU312718 | Honduras | 2003 | Lloyd et al. 2008 |
| EU312719 | Honduras | 2003 | Lloyd et al. 2008 |
| EU312720 | Honduras | 2003 | Lloyd et al. 2008 |
| EU312721 | Honduras | 2003 | Lloyd et al. 2008 |
| EU312722 | Honduras | 2003 | Lloyd et al. 2008 |
| EU312723 | Honduras | 2003 | Lloyd et al. 2008 |
| EU312724 | Honduras | 2003 | Lloyd et al. 2008 |
| EU312725 | Honduras | 2003 | Lloyd et al. 2008 |
| EU312726 | Honduras | 2003 | Lloyd et al. 2008 |
| EU312727 | Honduras | 2003 | Lloyd et al. 2008 |
| EU312728 | Honduras | 2003 | Lloyd et al. 2008 |
| EU312729 | Honduras | 2003 | Lloyd et al. 2008 |
| EU312730 | Honduras | 2003 | Lloyd et al. 2008 |
| EU312731 | Honduras | 2003 | Lloyd et al. 2008 |
| EU312732 | Honduras | 2003 | Lloyd et al. 2008 |
| EU312733 | Honduras | 2003 | Lloyd et al. 2008 |
| EU312734 | Honduras | 2003 | Lloyd et al. 2008 |
| EU312735 | Honduras | 2003 | Lloyd et al. 2008 |
| EU312736 | Honduras | 2003 | Lloyd et al. 2008 |
| EU312737 | Honduras | 2003 | Lloyd et al. 2008 |
| EU312738 | Honduras | 2003 | Lloyd et al. 2008 |
| EU312739 | Honduras | 2003 | Lloyd et al. 2008 |
| EU312740 | Honduras | 2003 | Lloyd et al. 2008 |
| EU312741 | Honduras | 2003 | Lloyd et al. 2008 |
| EU312742 | Honduras | 2003 | Lloyd et al. 2008 |
| EU312743 | Honduras | 2003 | Lloyd et al. 2008 |
| EU312744 | Honduras | 2003 | Lloyd et al. 2008 |
| EU312745 | Honduras | 2003 | Lloyd et al. 2008 |
| EU312746 | Honduras | 2003 | Lloyd et al. 2008 |
| EU312747 | Honduras | 2003 | Lloyd et al. 2008 |
| EU312748 | Honduras | 2003 | Lloyd et al. 2008 |
| EU312749 | Honduras | 2003 | Lloyd et al. 2008 |
| EU312750 | Honduras | 2003 | Lloyd et al. 2008 |
| EU312751 | Honduras | 2003 | Lloyd et al. 2008 |
| EU312752 | Honduras | 2003 | Lloyd et al. 2008 |
| EU312753 | Honduras | 2003 | Lloyd et al. 2008 |
| EU312754 | Honduras | 2003 | Lloyd et al. 2008 |
| EU312755 | Honduras | 2003 | Lloyd et al. 2008 |
| EU312756 | Honduras | 2003 | Lloyd et al. 2008 |
| EU312757 | Honduras | 2003 | Lloyd et al. 2008 |
| EU312758 | Honduras | 2003 | Lloyd et al. 2008 |
| EU312759 | Honduras | 2003 | Lloyd et al. 2008 |
| EU312760 | Honduras | 2003 | Lloyd et al. 2008 |
| EU312761 | Honduras | 2003 | Lloyd et al. 2008 |
| EU312762 | Honduras | 2003 | Lloyd et al. 2008 |
| EU312763 | Honduras | 2003 | Lloyd et al. 2008 |
| EU312764 | Honduras | 2003 | Lloyd et al. 2008 |
| EU312765 | Honduras | 2003 | Lloyd et al. 2008 |
| EU312766 | Honduras | 2003 | Lloyd et al. 2008 |
| EU312767 | Honduras | 2003 | Lloyd et al. 2008 |
| EU312768 | Honduras | 2003 | Lloyd et al. 2008 |
| EU312769 | Honduras | 2003 | Lloyd et al. 2008 |
| EU312770 | Honduras | 2003 | Lloyd et al. 2008 |
| EU312771 | Honduras | 2003 | Lloyd et al. 2008 |
| EU312772 | Honduras | 2003 | Lloyd et al. 2008 |
| EU312773 | Honduras | 2003 | Lloyd et al. 2008 |
| EU312774 | Honduras | 2003 | Lloyd et al. 2008 |
| EU312775 | Honduras | 2003 | Lloyd et al. 2008 |
| EU312776 | Honduras | 2003 | Lloyd et al. 2008 |
| EU312777 | Honduras | 2003 | Lloyd et al. 2008 |
| EU312778 | Honduras | 2003 | Lloyd et al. 2008 |
| EU312779 | Honduras | 2003 | Lloyd et al. 2008 |
| EU312780 | Honduras | 2003 | Lloyd et al. 2008 |
| EU312781 | Honduras | 2003 | Lloyd et al. 2008 |
| EU312782 | Honduras | 2003 | Lloyd et al. 2008 |
| EU312783 | Honduras | 2003 | Lloyd et al. 2008 |
| EU312785 | Honduras | 2003 | Lloyd et al. 2008 |
| EU312786 | Honduras | 2003 | Lloyd et al. 2008 |
| EU312787 | Honduras | 2003 | Lloyd et al. 2008 |
| EU312788 | Honduras | 2003 | Lloyd et al. 2008 |
| EU312789 | Honduras | 2003 | Lloyd et al. 2008 |
| EU312790 | Honduras | 2003 | Lloyd et al. 2008 |
| EU312791 | Honduras | 2003 | Lloyd et al. 2008 |
| EU312792 | Honduras | 2003 | Lloyd et al. 2008 |
| EU312793 | Honduras | 2003 | Lloyd et al. 2008 |
| EU312794 | Honduras | 2003 | Lloyd et al. 2008 |
| EU312795 | Honduras | 2003 | Lloyd et al. 2008 |
| EU312797 | Honduras | 2003 | Lloyd et al. 2008 |
| EU312798 | Honduras | 2003 | Lloyd et al. 2008 |
| EU312799 | Honduras | 2003 | Lloyd et al. 2008 |
| EU312800 | Honduras | 2003 | Lloyd et al. 2008 |
| EU312801 | Honduras | 2003 | Lloyd et al. 2008 |
| EU312802 | Honduras | 2003 | Lloyd et al. 2008 |
| EU312803 | Honduras | 2003 | Lloyd et al. 2008 |
| EU312804 | Honduras | 2003 | Lloyd et al. 2008 |
| EU312805 | Honduras | 2003 | Lloyd et al. 2008 |
| EU312806 | Honduras | 2003 | Lloyd et al. 2008 |
| EU312807 | Honduras | 2003 | Lloyd et al. 2008 |
| EU312808 | Honduras | 2003 | Lloyd et al. 2008 |
| EU312809 | Honduras | 2003 | Lloyd et al. 2008 |
| EU312810 | Honduras | 2003 | Lloyd et al. 2008 |
| EU312811 | Honduras | 2003 | Lloyd et al. 2008 |
| EU312812 | Honduras | 2003 | Lloyd et al. 2008 |
| EU312813 | Honduras | 2003 | Lloyd et al. 2008 |
| EU312814 | Honduras | 2003 | Lloyd et al. 2008 |
| EU312815 | Honduras | 2003 | Lloyd et al. 2008 |
| EU312816 | Honduras | 2003 | Lloyd et al. 2008 |
| EU312817 | Honduras | 2003 | Lloyd et al. 2008 |
| EU312818 | Honduras | 2003 | Lloyd et al. 2008 |
| EU312819 | Honduras | 2003 | Lloyd et al. 2008 |
| EU312820 | Honduras | 2003 | Lloyd et al. 2008 |
| EU312821 | Honduras | 2003 | Lloyd et al. 2008 |
| EU312822 | Honduras | 2003 | Lloyd et al. 2008 |
| EU312823 | Honduras | 2003 | Lloyd et al. 2008 |
| EU312824 | Honduras | 2003 | Lloyd et al. 2008 |
| EU312825 | Honduras | 2003 | Lloyd et al. 2008 |
| EU312826 | Honduras | 2003 | Lloyd et al. 2008 |
| EU312827 | Honduras | 2003 | Lloyd et al. 2008 |
| EU312828 | Honduras | 2003 | Lloyd et al. 2008 |
| EU312829 | Honduras | 2003 | Lloyd et al. 2008 |
| EU312830 | Honduras | 2003 | Lloyd et al. 2008 |
| EU312831 | Honduras | 2003 | Lloyd et al. 2008 |
| EU312832 | Honduras | 2003 | Lloyd et al. 2008 |
| EU312833 | Honduras | 2003 | Lloyd et al. 2008 |
| EU312834 | Honduras | 2003 | Lloyd et al. 2008 |
| EU312835 | Honduras | 2003 | Lloyd et al. 2008 |
| EU312836 | Honduras | 2003 | Lloyd et al. 2008 |
| EU312837 | Honduras | 2003 | Lloyd et al. 2008 |
| EU312838 | Honduras | 2003 | Lloyd et al. 2008 |
| EU312839 | Honduras | 2003 | Lloyd et al. 2008 |
| EU312840 | Honduras | 2003 | Lloyd et al. 2008 |
| EU312841 | Honduras | 2003 | Lloyd et al. 2008 |
| EU312842 | Honduras | 2003 | Lloyd et al. 2008 |
| EU312843 | Honduras | 2003 | Lloyd et al. 2008 |
| EU312844 | Honduras | 2003 | Lloyd et al. 2008 |
| EU312845 | Honduras | 2003 | Lloyd et al. 2008 |
| EU312846 | Honduras | 2003 | Lloyd et al. 2008 |
| EU312847 | Honduras | 2003 | Lloyd et al. 2008 |
| EU312848 | Honduras | 2003 | Lloyd et al. 2008 |
| EU312849 | Honduras | 2003 | Lloyd et al. 2008 |
| EU312850 | Honduras | 2003 | Lloyd et al. 2008 |
| EU312851 | Honduras | 2003 | Lloyd et al. 2008 |
| EU312852 | Honduras | 2003 | Lloyd et al. 2008 |
| EU312853 | Honduras | 2003 | Lloyd et al. 2008 |
| EU312854 | Honduras | 2003 | Lloyd et al. 2008 |
| EU312855 | Honduras | 2003 | Lloyd et al. 2008 |
| EU312856 | Honduras | 2003 | Lloyd et al. 2008 |
| EU312857 | Honduras | 2003 | Lloyd et al. 2008 |
| EU312858 | Honduras | 2003 | Lloyd et al. 2008 |
| EU312859 | Honduras | 2003 | Lloyd et al. 2008 |
| EU312860 | Honduras | 2003 | Lloyd et al. 2008 |
| EU312861 | Honduras | 2003 | Lloyd et al. 2008 |
| EU312862 | Honduras | 2003 | Lloyd et al. 2008 |
| EU312863 | Honduras | 2003 | Lloyd et al. 2008 |
| EU312864 | Honduras | 2003 | Lloyd et al. 2008 |
| EU312865 | Honduras | 2003 | Lloyd et al. 2008 |
| EU312866 | Honduras | 2003 | Lloyd et al. 2008 |
| EU312867 | Honduras | 2003 | Lloyd et al. 2008 |
| EU312868 | Honduras | 2003 | Lloyd et al. 2008 |
| EU312869 | Honduras | 2003 | Lloyd et al. 2008 |
| EU312870 | Honduras | 2003 | Lloyd et al. 2008 |
| EU312871 | Honduras | 2003 | Lloyd et al. 2008 |
| EU312872 | Honduras | 2003 | Lloyd et al. 2008 |
| EU312873 | Honduras | 2003 | Lloyd et al. 2008 |
| EU312874 | Honduras | 2003 | Lloyd et al. 2008 |
| EU312875 | Honduras | 2003 | Lloyd et al. 2008 |
| EU312876 | Honduras | 2003 | Lloyd et al. 2008 |
| EU312877 | Honduras | 2003 | Lloyd et al. 2008 |
| EU312878 | Honduras | 2003 | Lloyd et al. 2008 |
| EU312879 | Honduras | 2003 | Lloyd et al. 2008 |
| EU312880 | Honduras | 2003 | Lloyd et al. 2008 |
| EU312881 | Honduras | 2003 | Lloyd et al. 2008 |
| EU312882 | Honduras | 2003 | Lloyd et al. 2008 |
| EU312883 | Honduras | 2003 | Lloyd et al. 2008 |
| EU312884 | Honduras | 2003 | Lloyd et al. 2008 |
| EU312885 | Honduras | 2003 | Lloyd et al. 2008 |
| EU312886 | Honduras | 2003 | Lloyd et al. 2008 |
| EU312887 | Honduras | 2003 | Lloyd et al. 2008 |
| EU312888 | Honduras | 2003 | Lloyd et al. 2008 |
| EU312889 | Honduras | 2003 | Lloyd et al. 2008 |
| EU312890 | Honduras | 2003 | Lloyd et al. 2008 |
| EU312891 | Honduras | 2003 | Lloyd et al. 2008 |
| EU312892 | Honduras | 2003 | Lloyd et al. 2008 |
| EU312893 | Honduras | 2003 | Lloyd et al. 2008 |
| EU312894 | Honduras | 2003 | Lloyd et al. 2008 |
| EU312895 | Honduras | 2003 | Lloyd et al. 2008 |
| EU312896 | Honduras | 2003 | Lloyd et al. 2008 |
| EU312897 | Honduras | 2003 | Lloyd et al. 2008 |
| EU312898 | Honduras | 2003 | Lloyd et al. 2008 |
| EU312899 | Honduras | 2003 | Lloyd et al. 2008 |
| EU312900 | Honduras | 2003 | Lloyd et al. 2008 |
| EU312901 | Honduras | 2003 | Lloyd et al. 2008 |
| EU312902 | Honduras | 2003 | Lloyd et al. 2008 |
| EU312903 | Honduras | 2003 | Lloyd et al. 2008 |
| EU312904 | Honduras | 2003 | Lloyd et al. 2008 |
| EU312905 | Honduras | 2003 | Lloyd et al. 2008 |
| EU312906 | Honduras | 2003 | Lloyd et al. 2008 |
| EU312907 | Honduras | 2003 | Lloyd et al. 2008 |
| EU312908 | Honduras | 2003 | Lloyd et al. 2008 |
| EU312909 | Honduras | 2003 | Lloyd et al. 2008 |
| EU312910 | Honduras | 2003 | Lloyd et al. 2008 |
| EU312911 | Honduras | 2003 | Lloyd et al. 2008 |
| EU312912 | Honduras | 2003 | Lloyd et al. 2008 |
| EU312913 | Honduras | 2003 | Lloyd et al. 2008 |
| EU312914 | Honduras | 2003 | Lloyd et al. 2008 |
| EU312915 | Honduras | 2003 | Lloyd et al. 2008 |
| EU312916 | Honduras | 2003 | Lloyd et al. 2008 |
| EU312917 | Honduras | 2003 | Lloyd et al. 2008 |
| EU312918 | Honduras | 2003 | Lloyd et al. 2008 |
| EU312919 | Honduras | 2003 | Lloyd et al. 2008 |
| EU312920 | Honduras | 2003 | Lloyd et al. 2008 |
| EU312921 | Honduras | 2003 | Lloyd et al. 2008 |
| EU312922 | Honduras | 2003 | Lloyd et al. 2008 |
| EU312923 | Honduras | 2003 | Lloyd et al. 2008 |
| EU312924 | Honduras | 2003 | Lloyd et al. 2008 |
| EU312925 | Honduras | 2003 | Lloyd et al. 2008 |
| EU312926 | Honduras | 2003 | Lloyd et al. 2008 |
| EU312927 | Honduras | 2003 | Lloyd et al. 2008 |
| EU312928 | Honduras | 2003 | Lloyd et al. 2008 |
| EU312929 | Honduras | 2003 | Lloyd et al. 2008 |
| EU312930 | Honduras | 2003 | Lloyd et al. 2008 |
| EU312931 | Honduras | 2003 | Lloyd et al. 2008 |
| EU312932 | Honduras | 2003 | Lloyd et al. 2008 |
| EU312933 | Honduras | 2003 | Lloyd et al. 2008 |
| EU312934 | Honduras | 2003 | Lloyd et al. 2008 |
| EU312935 | Honduras | 2003 | Lloyd et al. 2008 |
| EU312936 | Honduras | 2003 | Lloyd et al. 2008 |
| EU312937 | Honduras | 2003 | Lloyd et al. 2008 |
| EU312938 | Honduras | 2003 | Lloyd et al. 2008 |
| EU312939 | Honduras | 2003 | Lloyd et al. 2008 |
| EU312940 | Honduras | 2003 | Lloyd et al. 2008 |
| EU312941 | Honduras | 2003 | Lloyd et al. 2008 |
| EU312942 | Honduras | 2003 | Lloyd et al. 2008 |
| EU312943 | Honduras | 2003 | Lloyd et al. 2008 |
| EU312944 | Honduras | 2003 | Lloyd et al. 2008 |
| EU312945 | Honduras | 2003 | Lloyd et al. 2008 |
| EU312946 | Honduras | 2003 | Lloyd et al. 2008 |
| EU312947 | Honduras | 2003 | Lloyd et al. 2008 |
| EU312948 | Honduras | 2003 | Lloyd et al. 2008 |
| EU312949 | Honduras | 2003 | Lloyd et al. 2008 |
| EU312950 | Honduras | 2003 | Lloyd et al. 2008 |
| EU312951 | Honduras | 2003 | Lloyd et al. 2008 |
| EU312952 | Honduras | 2003 | Lloyd et al. 2008 |
| EU312953 | Honduras | 2003 | Lloyd et al. 2008 |
| EU312954 | Honduras | 2003 | Lloyd et al. 2008 |
| EU312955 | Honduras | 2003 | Lloyd et al. 2008 |
| EU312956 | Honduras | 2003 | Lloyd et al. 2008 |
| EU312957 | Honduras | 2003 | Lloyd et al. 2008 |
| EU312958 | Honduras | 2003 | Lloyd et al. 2008 |
| EU312959 | Honduras | 2003 | Lloyd et al. 2008 |
| EU312960 | Honduras | 2003 | Lloyd et al. 2008 |
| EU312961 | Honduras | 2003 | Lloyd et al. 2008 |
| EU312962 | Honduras | 2003 | Lloyd et al. 2008 |
| EU312963 | Honduras | 2003 | Lloyd et al. 2008 |
| EU312964 | Honduras | 2003 | Lloyd et al. 2008 |
| EU312965 | Honduras | 2003 | Lloyd et al. 2008 |
| EU312966 | Honduras | 2003 | Lloyd et al. 2008 |
| EU312967 | Honduras | 2003 | Lloyd et al. 2008 |
| EU312968 | Honduras | 2003 | Lloyd et al. 2008 |
| EU312969 | Honduras | 2003 | Lloyd et al. 2008 |
| EU312970 | Honduras | 2003 | Lloyd et al. 2008 |
| EU312971 | Honduras | 2003 | Lloyd et al. 2008 |
| EU312972 | Honduras | 2003 | Lloyd et al. 2008 |
| FJ800379 | Honduras | 2004 | Murillo et al. 2010 |
| FJ800380 | Honduras | 2005 | Murillo et al. 2010 |
| FJ800381 | Honduras | 2004 | Murillo et al. 2010 |
| FJ800382 | Honduras | 2004 | Murillo et al. 2010 |
| FJ800383 | Honduras | 2005 | Murillo et al. 2010 |
| FJ800384 | Honduras | 2005 | Murillo et al. 2010 |
| FJ800385 | Honduras | 2005 | Murillo et al. 2010 |
| FJ800386 | Honduras | 2005 | Murillo et al. 2010 |
| FJ800387 | Honduras | 2005 | Murillo et al. 2010 |
| FJ800388 | Honduras | 2005 | Murillo et al. 2010 |
| FJ800389 | Honduras | 2005 | Murillo et al. 2010 |
| FJ800390 | Honduras | 2004 | Murillo et al. 2010 |
| FJ800391 | Honduras | 2005 | Murillo et al. 2010 |
| FJ800392 | Honduras | 2005 | Murillo et al. 2010 |
| FJ800393 | Honduras | 2005 | Murillo et al. 2010 |
| FJ800394 | Honduras | 2005 | Murillo et al. 2010 |
| FJ800395 | Honduras | 2005 | Murillo et al. 2010 |
| FJ800396 | Honduras | 2005 | Murillo et al. 2010 |
| FJ800397 | Honduras | 2005 | Murillo et al. 2010 |
| FJ800398 | Honduras | 2005 | Murillo et al. 2010 |
| FJ800399 | Honduras | 2005 | Murillo et al. 2010 |
| FJ800400 | Honduras | 2005 | Murillo et al. 2010 |
| FJ800401 | Honduras | 2005 | Murillo et al. 2010 |
| FJ800402 | Honduras | 2005 | Murillo et al. 2010 |
| FJ800403 | Honduras | 2005 | Murillo et al. 2010 |
| FJ800404 | Honduras | 2005 | Murillo et al. 2010 |
| FJ800405 | Honduras | 2005 | Murillo et al. 2010 |
| FJ800406 | Honduras | 2005 | Murillo et al. 2010 |
| FJ800407 | Honduras | 2005 | Murillo et al. 2010 |
| FJ800408 | Honduras | 2005 | Murillo et al. 2010 |
| FJ800409 | Honduras | 2005 | Murillo et al. 2010 |
| FJ800410 | Honduras | 2005 | Murillo et al. 2010 |
| FJ800400 | Honduras | 2005 | Murillo et al. 2010 |
| FJ800412 | Honduras | 2005 | Murillo et al. 2010 |
| FJ800413 | Honduras | 2005 | Murillo et al. 2010 |
| FJ800414 | Honduras | 2005 | Murillo et al. 2010 |
| FJ800415 | Honduras | 2005 | Murillo et al. 2010 |
| FJ800416 | Honduras | 2005 | Murillo et al. 2010 |
| FJ800417 | Honduras | 2005 | Murillo et al. 2010 |
| FJ800418 | Honduras | 2005 | Murillo et al. 2010 |
| FJ800419 | Honduras | 2005 | Murillo et al. 2010 |
| FJ800420 | Honduras | 2006 | Murillo et al. 2010 |
| FJ800421 | Honduras | 2006 | Murillo et al. 2010 |
| FJ800422 | Honduras | 2006 | Murillo et al. 2010 |
| FJ800423 | Honduras | 2006 | Murillo et al. 2010 |
| FJ800424 | Honduras | 2006 | Murillo et al. 2010 |
| FJ800425 | Honduras | 2006 | Murillo et al. 2010 |
| FJ800426 | Honduras | 2006 | Murillo et al. 2010 |
| FJ800427 | Honduras | 2006 | Murillo et al. 2010 |
| FJ800428 | Honduras | 2006 | Murillo et al. 2010 |
| FJ800429 | Honduras | 2006 | Murillo et al. 2010 |
| FJ800430 | Honduras | 2006 | Murillo et al. 2010 |
| FJ800431 | Honduras | 2006 | Murillo et al. 2010 |
| FJ800432 | Honduras | 2006 | Murillo et al. 2010 |
| FJ800433 | Honduras | 2006 | Murillo et al. 2010 |
| FJ800434 | Honduras | 2006 | Murillo et al. 2010 |
| FJ800435 | Honduras | 2006 | Murillo et al. 2010 |
| FJ800436 | Honduras | 2006 | Murillo et al. 2010 |
| FJ800437 | Honduras | 2006 | Murillo et al. 2010 |
| FJ800438 | Honduras | 2006 | Murillo et al. 2010 |
| FJ800439 | Honduras | 2006 | Murillo et al. 2010 |
| FJ800440 | Honduras | 2006 | Murillo et al. 2010 |
| FJ800441 | Honduras | 2006 | Murillo et al. 2010 |
| FJ800442 | Honduras | 2006 | Murillo et al. 2010 |
| FJ800443 | Honduras | 2006 | Murillo et al. 2010 |
| FJ800444 | Honduras | 2006 | Murillo et al. 2010 |
| FJ800445 | Honduras | 2006 | Murillo et al. 2010 |
| FJ800446 | Honduras | 2006 | Murillo et al. 2010 |
| FJ800447 | Honduras | 2006 | Murillo et al. 2010 |
| FJ800448 | Honduras | 2006 | Murillo et al. 2010 |
| FJ800449 | Honduras | 2006 | Murillo et al. 2010 |
| FJ800450 | Honduras | 2006 | Murillo et al. 2010 |
| FJ800451 | Honduras | 2006 | Murillo et al. 2010 |
| FJ800452 | Honduras | 2006 | Murillo et al. 2010 |
| FJ800453 | Honduras | 2006 | Murillo et al. 2010 |
| FJ800454 | Honduras | 2006 | Murillo et al. 2010 |
| FJ800455 | Honduras | 2006 | Murillo et al. 2010 |
| FJ800456 | Honduras | 2006 | Murillo et al. 2010 |
| FJ800457 | Honduras | 2006 | Murillo et al. 2010 |
| FJ800458 | Honduras | 2006 | Murillo et al. 2010 |
| FJ800459 | Honduras | 2006 | Murillo et al. 2010 |
| FJ800460 | Honduras | 2006 | Murillo et al. 2010 |
| FJ800461 | Honduras | 2006 | Murillo et al. 2010 |
| FJ800462 | Honduras | 2006 | Murillo et al. 2010 |
| FJ800463 | Honduras | 2006 | Murillo et al. 2010 |
| FJ800464 | Honduras | 2006 | Murillo et al. 2010 |
| FJ800465 | Honduras | 2006 | Murillo et al. 2010 |
| FJ800466 | Honduras | 2006 | Murillo et al. 2010 |
| FJ800467 | Honduras | 2006 | Murillo et al. 2010 |
| FJ800468 | Honduras | 2006 | Murillo et al. 2010 |
| FJ800469 | Honduras | 2006 | Murillo et al. 2010 |
| FJ800470 | Honduras | 2006 | Murillo et al. 2010 |
| FJ800471 | Honduras | 2006 | Murillo et al. 2010 |
| FJ800472 | Honduras | 2006 | Murillo et al. 2010 |
| FJ800473 | Honduras | 2006 | Murillo et al. 2010 |
| FJ800474 | Honduras | 2006 | Murillo et al. 2010 |
| FJ800475 | Honduras | 2006 | Murillo et al. 2010 |
| FJ800476 | Honduras | 2006 | Murillo et al. 2010 |
| FJ800477 | Honduras | 2006 | Murillo et al. 2010 |
| FJ800478 | Honduras | 2006 | Murillo et al. 2010 |
| FJ800479 | Honduras | 2006 | Murillo et al. 2010 |
| FJ800480 | Honduras | 2006 | Murillo et al. 2010 |
| FJ800481 | Honduras | 2006 | Murillo et al. 2010 |
| FJ800482 | Honduras | 2006 | Murillo et al. 2010 |
| FJ800483 | Honduras | 2006 | Murillo et al. 2010 |
| FJ800484 | Honduras | 2007 | Murillo et al. 2010 |
| FJ800485 | Honduras | 2007 | Murillo et al. 2010 |
| FJ800486 | Honduras | 2007 | Murillo et al. 2010 |
| FJ800487 | Honduras | 2007 | Murillo et al. 2010 |
| FJ800488 | Honduras | 2007 | Murillo et al. 2010 |
| FJ800489 | Honduras | 2007 | Murillo et al. 2010 |
| FJ800490 | Honduras | 2007 | Murillo et al. 2010 |
| FJ800491 | Honduras | 2007 | Murillo et al. 2010 |
| FJ800492 | Honduras | 2007 | Murillo et al. 2010 |
| FJ800493 | Honduras | 2007 | Murillo et al. 2010 |
| FJ800494 | Honduras | 2007 | Murillo et al. 2010 |
| FJ800495 | Honduras | 2007 | Murillo et al. 2010 |
| FJ800496 | Honduras | 2007 | Murillo et al. 2010 |
| FJ800497 | Honduras | 2007 | Murillo et al. 2010 |
| FJ800498 | Honduras | 2007 | Murillo et al. 2010 |
| FJ800499 | Honduras | 2007 | Murillo et al. 2010 |
| FJ800500 | Honduras | 2007 | Murillo et al. 2010 |
| FJ800501 | Honduras | 2007 | Murillo et al. 2010 |
| FJ800502 | Honduras | 2007 | Murillo et al. 2010 |
| FJ800503 | Honduras | 2007 | Murillo et al. 2010 |
| FJ800504 | Honduras | 2007 | Murillo et al. 2010 |
| FJ800505 | Honduras | 2007 | Murillo et al. 2010 |
| FJ823645 | Honduras | 2004 | Murillo et al. 2010 |
| FJ823646 | Honduras | 2005 | Murillo et al. 2010 |
| FJ823647 | Honduras | 2005 | Murillo et al. 2010 |
| FJ823648 | Honduras | 2005 | Murillo et al. 2010 |
| FJ823649 | Honduras | 2004 | Murillo et al. 2010 |
| FJ823650 | Honduras | 2004 | Murillo et al. 2010 |
| FJ823651 | Honduras | 2004 | Murillo et al. 2010 |
| FJ823652 | Honduras | 2004 | Murillo et al. 2010 |
| FJ823653 | Honduras | 2004 | Murillo et al. 2010 |
| FJ823654 | Honduras | 2004 | Murillo et al. 2010 |
| FJ823655 | Honduras | 2004 | Murillo et al. 2010 |
| FJ823656 | Honduras | 2004 | Murillo et al. 2010 |
| FJ823657 | Honduras | 2004 | Murillo et al. 2010 |
| HQ010598 | Honduras | 2010 | Holguin et al. 2011 |
| HQ010599 | Honduras | 2010 | Holguin et al. 2011 |
| HQ010600 | Honduras | 2010 | Holguin et al. 2011 |
| HQ010601 | Honduras | 2010 | Holguin et al. 2011 |
| HQ010602 | Honduras | 2010 | Holguin et al. 2011 |
| HQ010603 | Honduras | 2010 | Holguin et al. 2011 |
| HQ010604 | Honduras | 2010 | Holguin et al. 2011 |
| HQ010605 | Honduras | 2010 | Holguin et al. 2011 |
| HQ010606 | Honduras | 2010 | Holguin et al. 2011 |
| HQ010607 | Honduras | 2010 | Holguin et al. 2011 |
| HQ010608 | Honduras | 2010 | Holguin et al. 2011 |
| HQ010609 | Honduras | 2010 | Holguin et al. 2011 |
| HQ010610 | Honduras | 2010 | Holguin et al. 2011 |
| HQ010611 | Honduras | 2010 | Holguin et al. 2011 |
| HQ010612 | Honduras | 2010 | Holguin et al. 2011 |
| HQ010613 | Honduras | 2010 | Holguin et al. 2011 |
| HQ010614 | Honduras | 2010 | Holguin et al. 2011 |
| HQ010615 | Honduras | 2010 | Holguin et al. 2011 |
| HQ010616 | Honduras | 2010 | Holguin et al. 2011 |
| HQ010617 | Honduras | 2010 | Holguin et al. 2011 |
| HQ010618 | Honduras | 2010 | Holguin et al. 2011 |
| HQ010619 | Honduras | 2010 | Holguin et al. 2011 |
| HQ010620 | Honduras | 2010 | Holguin et al. 2011 |
| HQ010621 | Honduras | 2010 | Holguin et al. 2011 |
| HQ010622 | Honduras | 2010 | Holguin et al. 2011 |
| HQ010623 | Honduras | 2010 | Holguin et al. 2011 |
| HQ010624 | Honduras | 2010 | Holguin et al. 2011 |
| HQ010625 | Honduras | 2010 | Holguin et al. 2011 |
| HQ010626 | Honduras | 2010 | Holguin et al. 2011 |
| HQ010627 | Honduras | 2010 | Holguin et al. 2011 |
| HQ010628 | Honduras | 2010 | Holguin et al. 2011 |
| HQ010629 | Honduras | 2010 | Holguin et al. 2011 |
| HQ010630 | Honduras | 2010 | Holguin et al. 2011 |
| HQ010631 | Honduras | 2010 | Holguin et al. 2011 |
| HQ010632 | Honduras | 2010 | Holguin et al. 2011 |
| HQ010633 | Honduras | 2010 | Holguin et al. 2011 |
| HQ010634 | Honduras | 2010 | Holguin et al. 2011 |
| HQ010635 | Honduras | 2010 | Holguin et al. 2011 |
| HQ010636 | Honduras | 2010 | Holguin et al. 2011 |
| HQ010637 | Honduras | 2010 | Holguin et al. 2011 |
| HQ010638 | Honduras | 2010 | Holguin et al. 2011 |
| HQ010639 | Honduras | 2010 | Holguin et al. 2011 |
| HQ010640 | Honduras | 2010 | Holguin et al. 2011 |
| HQ010641 | Honduras | 2010 | Holguin et al. 2011 |
| HQ010642 | Honduras | 2010 | Holguin et al. 2011 |
| HQ010643 | Honduras | 2010 | Holguin et al. 2011 |
| HQ010644 | Honduras | 2010 | Holguin et al. 2011 |
| HQ010645 | Honduras | 2010 | Holguin et al. 2011 |
| HQ010646 | Honduras | 2010 | Holguin et al. 2011 |
| HQ010647 | Honduras | 2010 | Holguin et al. 2011 |
| HQ010648 | Honduras | 2010 | Holguin et al. 2011 |
| HQ010649 | Honduras | 2010 | Holguin et al. 2011 |
| HQ599240 | Honduras | 2002 | Parham et al. 2011 |
| HQ599241 | Honduras | 2003 | Parham et al. 2011 |
| HQ599242 | Honduras | 2003 | Parham et al. 2011 |
| HQ599243 | Honduras | 2003 | Parham et al. 2011 |
| HQ599245 | Honduras | 2004 | Parham et al. 2011 |
| HQ599246 | Honduras | 2004 | Parham et al. 2011 |
| HQ842974 | Honduras | 2004 | Parham et al. 2011 |
| HQ842975 | Honduras | 2002 | Parham et al. 2011 |
| HQ842976 | Honduras | 2002 | Parham et al. 2011 |
| HQ842977 | Honduras | 2003 | Parham et al. 2011 |
| HQ842978 | Honduras | 2003 | Parham et al. 2011 |
| HQ842979 | Honduras | 2003 | Parham et al. 2011 |
| HQ842980 | Honduras | 2002 | Parham et al. 2011 |
| HQ842981 | Honduras | 2002 | Parham et al. 2011 |
| HQ842982 | Honduras | 2002 | Parham et al. 2011 |
| HQ842983 | Honduras | 2002 | Parham et al. 2011 |
| HQ842984 | Honduras | 2001 | Parham et al. 2011 |
| HQ842985 | Honduras | 2002 | Parham et al. 2011 |
| HQ842986 | Honduras | 2003 | Parham et al. 2011 |
| HQ842987 | Honduras | 2002 | Parham et al. 2011 |
| HQ842988 | Honduras | 2002 | Parham et al. 2011 |
| HQ842989 | Honduras | 2003 | Parham et al. 2011 |
| HQ842990 | Honduras | 2003 | Parham et al. 2011 |
| HQ842991 | Honduras | 2004 | Parham et al. 2011 |
| HQ842992 | Honduras | 2004 | Parham et al. 2011 |
| HQ842993 | Honduras | 2001 | Parham et al. 2011 |
| HQ842994 | Honduras | 2001 | Parham et al. 2011 |
| HQ842995 | Honduras | 2001 | Parham et al. 2011 |
| HQ842996 | Honduras | 2001 | Parham et al. 2011 |
| HQ842997 | Honduras | 2001 | Parham et al. 2011 |
| HQ842998 | Honduras | 2002 | Parham et al. 2011 |
| HQ842999 | Honduras | 2002 | Parham et al. 2011 |
| HQ843000 | Honduras | 2002 | Parham et al. 2011 |
| HQ843001 | Honduras | 2002 | Parham et al. 2011 |
| HQ843002 | Honduras | 2002 | Parham et al. 2011 |
| HQ843003 | Honduras | 2003 | Parham et al. 2011 |
| HQ843004 | Honduras | 2003 | Parham et al. 2011 |
| HQ843005 | Honduras | 2003 | Parham et al. 2011 |
| HQ843006 | Honduras | 2003 | Parham et al. 2011 |
| HQ843007 | Honduras | 2003 | Parham et al. 2011 |
| HQ843008 | Honduras | 2003 | Parham et al. 2011 |
| HQ843009 | Honduras | 2003 | Parham et al. 2011 |
| HQ843010 | Honduras | 2003 | Parham et al. 2011 |
| HQ843011 | Honduras | 2003 | Parham et al. 2011 |
| HQ843012 | Honduras | 2003 | Parham et al. 2011 |
| HQ843013 | Honduras | 2003 | Parham et al. 2011 |
| HQ843014 | Honduras | 2003 | Parham et al. 2011 |
| HQ843015 | Honduras | 2003 | Parham et al. 2011 |
| HQ843016 | Honduras | 2003 | Parham et al. 2011 |
| HQ843017 | Honduras | 2003 | Parham et al. 2011 |
| HQ843018 | Honduras | 2004 | Parham et al. 2011 |
| HQ843019 | Honduras | 2004 | Parham et al. 2011 |
| HQ843020 | Honduras | 2004 | Parham et al. 2011 |
| HQ843021 | Honduras | 2004 | Parham et al. 2011 |
|  |  |  |  |
| AY812748 | Mexico | 2001/2003 | Valle-Bahena et al. 2006 |
| AY812749 | Mexico | 2001/2003 | Valle-Bahena et al. 2006 |
| AY812750 | Mexico | 2001/2003 | Valle-Bahena et al. 2006 |
| AY926353 | Mexico | 2001/2003 | Valle-Bahena et al. 2006 |
| AY926354 | Mexico | 2001/2003 | Valle-Bahena et al. 2006 |
| AY926355 | Mexico | 2001/2003 | Valle-Bahena et al. 2006 |
| AY926356 | Mexico | 2001/2003 | Valle-Bahena et al. 2006 |
| AY926357 | Mexico | 2001/2003 | Valle-Bahena et al. 2006 |
| DQ150702 | Mexico | 2001/2003 | Valle-Bahena et al. 2006 |
| DQ150703 | Mexico | 2001/2003 | Valle-Bahena et al. 2006 |
| DQ150704 | Mexico | 2001/2003 | Valle-Bahena et al. 2006 |
| DQ150705 | Mexico | 2001/2003 | Valle-Bahena et al. 2006 |
| DQ150706 | Mexico | 2001/2003 | Valle-Bahena et al. 2006 |
| DQ150707 | Mexico | 2001/2003 | Valle-Bahena et al. 2006 |
| DQ150708 | Mexico | 2001/2003 | Valle-Bahena et al. 2006 |
| DQ150709 | Mexico | 2001/2003 | Valle-Bahena et al. 2006 |
| DQ150710 | Mexico | 2001/2003 | Valle-Bahena et al. 2006 |
| DQ150711 | Mexico | 2001/2003 | Valle-Bahena et al. 2006 |
| DQ150714 | Mexico | 2001/2003 | Valle-Bahena et al. 2006 |
| DQ150717 | Mexico | 2001/2003 | Valle-Bahena et al. 2006 |
| DQ150718 | Mexico | 2001/2003 | Valle-Bahena et al. 2006 |
| DQ150719 | Mexico | 2001/2003 | Valle-Bahena et al. 2006 |
| DQ150720 | Mexico | 2001/2003 | Valle-Bahena et al. 2006 |
| DQ150721 | Mexico | 2001/2003 | Valle-Bahena et al. 2006 |
| DQ150722 | Mexico | 2001/2003 | Valle-Bahena et al. 2006 |
| DQ150723 | Mexico | 2001/2003 | Valle-Bahena et al. 2006 |
| DQ150724 | Mexico | 2001/2003 | Valle-Bahena et al. 2006 |
| DQ150725 | Mexico | 2001/2003 | Valle-Bahena et al. 2006 |
| DQ150729 | Mexico | 2001/2003 | Valle-Bahena et al. 2006 |
| DQ150730 | Mexico | 2001/2003 | Valle-Bahena et al. 2006 |
| DQ518490 | Mexico | 2004 | Gomez-Carrillo et al. 2006 |
| DQ518491 | Mexico | 2004 | Gomez-Carrillo et al. 2006 |
| DQ518492 | Mexico | 2004 | Gomez-Carrillo et al. 2006 |
| DQ518493 | Mexico | 2004 | Gomez-Carrillo et al. 2006 |
| DQ518494 | Mexico | 2004 | Gomez-Carrillo et al. 2006 |
| DQ631415 | Mexico | 2005 | Eyzaguirre et al. 2007 |
| DQ631416 | Mexico | 2005 | Eyzaguirre et al. 2007 |
| DQ631417 | Mexico | 2005 | Eyzaguirre et al. 2007 |
| DQ631418 | Mexico | 2005 | Eyzaguirre et al. 2007 |
| DQ631419 | Mexico | 2005 | Eyzaguirre et al. 2007 |
| DQ631420 | Mexico | 2005 | Eyzaguirre et al. 2007 |
| DQ631421 | Mexico | 2005 | Eyzaguirre et al. 2007 |
| DQ631422 | Mexico | 2005 | Eyzaguirre et al. 2007 |
| DQ631423 | Mexico | 2005 | Eyzaguirre et al. 2007 |
| DQ631424 | Mexico | 2005 | Eyzaguirre et al. 2007 |
| DQ631425 | Mexico | 2005 | Eyzaguirre et al. 2007 |
|  |  |  |  |
| FJ713155 | Panama | 2005 | Ahumada-Ruiz et al. 2009 |
| FJ713156 | Panama | 2005 | Ahumada-Ruiz et al. 2009 |
| FJ713157 | Panama | 2005 | Ahumada-Ruiz et al. 2009 |
| FJ713158 | Panama | 2005 | Ahumada-Ruiz et al. 2009 |
| FJ713159 | Panama | 2005 | Ahumada-Ruiz et al. 2009 |
| FJ713160 | Panama | 2005 | Ahumada-Ruiz et al. 2009 |
| FJ713161 | Panama | 2005 | Ahumada-Ruiz et al. 2009 |
| FJ713162 | Panama | 2005 | Ahumada-Ruiz et al. 2009 |
| FJ713163 | Panama | 2005 | Ahumada-Ruiz et al. 2009 |
| FJ713164 | Panama | 2005 | Ahumada-Ruiz et al. 2009 |
| FJ713165 | Panama | 2005 | Ahumada-Ruiz et al. 2009 |
| FJ713166 | Panama | 2005 | Ahumada-Ruiz et al. 2009 |
| FJ713167 | Panama | 2005 | Ahumada-Ruiz et al. 2009 |
| FJ713168 | Panama | 2005 | Ahumada-Ruiz et al. 2009 |
| FJ713169 | Panama | 2005 | Ahumada-Ruiz et al. 2009 |
| FJ713170 | Panama | 2005 | Ahumada-Ruiz et al. 2009 |
| FJ713171 | Panama | 2005 | Ahumada-Ruiz et al. 2009 |
| FJ713172 | Panama | 2005 | Ahumada-Ruiz et al. 2009 |
| FJ713173 | Panama | 2005 | Ahumada-Ruiz et al. 2009 |
| FJ713174 | Panama | 2005 | Ahumada-Ruiz et al. 2009 |
| FJ713175 | Panama | 2005 | Ahumada-Ruiz et al. 2009 |
| FJ713176 | Panama | 2005 | Ahumada-Ruiz et al. 2009 |
| FJ713177 | Panama | 2005 | Ahumada-Ruiz et al. 2009 |
| FJ713178 | Panama | 2005 | Ahumada-Ruiz et al. 2009 |
| FJ713179 | Panama | 2005 | Ahumada-Ruiz et al. 2009 |
| FJ713180 | Panama | 2005 | Ahumada-Ruiz et al. 2009 |
| FJ713181 | Panama | 2005 | Ahumada-Ruiz et al. 2009 |
| FJ713182 | Panama | 2005 | Ahumada-Ruiz et al. 2009 |
| FJ713183 | Panama | 2005 | Ahumada-Ruiz et al. 2009 |
| FJ713184 | Panama | 2005 | Ahumada-Ruiz et al. 2009 |
| FJ713185 | Panama | 2005 | Ahumada-Ruiz et al. 2009 |
| FJ713186 | Panama | 2005 | Ahumada-Ruiz et al. 2009 |
| FJ713187 | Panama | 2005 | Ahumada-Ruiz et al. 2009 |
| FJ713188 | Panama | 2005 | Ahumada-Ruiz et al. 2009 |
| FJ713189 | Panama | 2005 | Ahumada-Ruiz et al. 2009 |
| FJ713190 | Panama | 2005 | Ahumada-Ruiz et al. 2009 |
| FJ713191 | Panama | 2005 | Ahumada-Ruiz et al. 2009 |
| FJ713192 | Panama | 2005 | Ahumada-Ruiz et al. 2009 |
| FJ713193 | Panama | 2005 | Ahumada-Ruiz et al. 2009 |
| FJ713194 | Panama | 2005 | Ahumada-Ruiz et al. 2009 |
| FJ713195 | Panama | 2005 | Ahumada-Ruiz et al. 2009 |
| FJ713196 | Panama | 2005 | Ahumada-Ruiz et al. 2009 |
| FJ713197 | Panama | 2005 | Ahumada-Ruiz et al. 2009 |
| FJ713198 | Panama | 2005 | Ahumada-Ruiz et al. 2009 |
| FJ713199 | Panama | 2005 | Ahumada-Ruiz et al. 2009 |
| FJ713200 | Panama | 2005 | Ahumada-Ruiz et al. 2009 |
| FJ713201 | Panama | 2005 | Ahumada-Ruiz et al. 2009 |
| FJ713202 | Panama | 2005 | Ahumada-Ruiz et al. 2009 |
| FJ713203 | Panama | 2005 | Ahumada-Ruiz et al. 2009 |
| FJ713204 | Panama | 2005 | Ahumada-Ruiz et al. 2009 |
| FJ713205 | Panama | 2005 | Ahumada-Ruiz et al. 2009 |
| FJ713206 | Panama | 2005 | Ahumada-Ruiz et al. 2009 |
| FJ713207 | Panama | 2005 | Ahumada-Ruiz et al. 2009 |
| FJ713208 | Panama | 2004 | Ahumada-Ruiz et al. 2009 |
| FJ713209 | Panama | 2004 | Ahumada-Ruiz et al. 2009 |
| FJ713210 | Panama | 2004 | Ahumada-Ruiz et al. 2009 |
| FJ713211 | Panama | 2004 | Ahumada-Ruiz et al. 2009 |
| FJ713212 | Panama | 2004 | Ahumada-Ruiz et al. 2009 |
| FJ713213 | Panama | 2004 | Ahumada-Ruiz et al. 2009 |
| FJ713214 | Panama | 2004 | Ahumada-Ruiz et al. 2009 |
| FJ713215 | Panama | 2004 | Ahumada-Ruiz et al. 2009 |
| FJ713216 | Panama | 2004 | Ahumada-Ruiz et al. 2009 |
| FJ713218 | Panama | 2004 | Ahumada-Ruiz et al. 2009 |
| FJ713219 | Panama | 2004 | Ahumada-Ruiz et al. 2009 |
| FJ713220 | Panama | 2004 | Ahumada-Ruiz et al. 2009 |
| FJ713221 | Panama | 2004 | Ahumada-Ruiz et al. 2009 |
| FJ713222 | Panama | 2004 | Ahumada-Ruiz et al. 2009 |
| FJ713223 | Panama | 2004 | Ahumada-Ruiz et al. 2009 |
| FJ713224 | Panama | 2004 | Ahumada-Ruiz et al. 2009 |
| FJ713225 | Panama | 2004 | Ahumada-Ruiz et al. 2009 |
| FJ713226 | Panama | 2004 | Ahumada-Ruiz et al. 2009 |
| FJ713227 | Panama | 2004 | Ahumada-Ruiz et al. 2009 |
| FJ713228 | Panama | 2004 | Ahumada-Ruiz et al. 2009 |
| FJ713229 | Panama | 2004 | Ahumada-Ruiz et al. 2009 |
| FJ713230 | Panama | 2004 | Ahumada-Ruiz et al. 2009 |
| FJ713231 | Panama | 2004 | Ahumada-Ruiz et al. 2009 |
| FJ713232 | Panama | 2004 | Ahumada-Ruiz et al. 2009 |
| FJ713234 | Panama | 2004 | Ahumada-Ruiz et al. 2009 |
| FJ713235 | Panama | 2004 | Ahumada-Ruiz et al. 2009 |
| FJ713236 | Panama | 2004 | Ahumada-Ruiz et al. 2009 |
| FJ713237 | Panama | 2004 | Ahumada-Ruiz et al. 2009 |
| FJ713238 | Panama | 2004 | Ahumada-Ruiz et al. 2009 |
| FJ713239 | Panama | 2004 | Ahumada-Ruiz et al. 2009 |
| FJ713240 | Panama | 2004 | Ahumada-Ruiz et al. 2009 |
| FJ713241 | Panama | 2004 | Ahumada-Ruiz et al. 2009 |
| FJ713242 | Panama | 2004 | Ahumada-Ruiz et al. 2009 |
| FJ713243 | Panama | 2004 | Ahumada-Ruiz et al. 2009 |
| FJ713244 | Panama | 2005 | Ahumada-Ruiz et al. 2009 |
| FJ713245 | Panama | 2005 | Ahumada-Ruiz et al. 2009 |
| FJ713246 | Panama | 2005 | Ahumada-Ruiz et al. 2009 |
| FJ713247 | Panama | 2005 | Ahumada-Ruiz et al. 2009 |
| FJ713248 | Panama | 2005 | Ahumada-Ruiz et al. 2009 |
| FJ713249 | Panama | 2005 | Ahumada-Ruiz et al. 2009 |
| FJ713250 | Panama | 2005 | Ahumada-Ruiz et al. 2009 |
| FJ713251 | Panama | 2005 | Ahumada-Ruiz et al. 2009 |
| FJ713252 | Panama | 2005 | Ahumada-Ruiz et al. 2009 |
| FJ713253 | Panama | 2005 | Ahumada-Ruiz et al. 2009 |
| FJ713254 | Panama | 2005 | Ahumada-Ruiz et al. 2009 |
| FJ713255 | Panama | 2005 | Ahumada-Ruiz et al. 2009 |
| FJ713256 | Panama | 2005 | Ahumada-Ruiz et al. 2009 |
| FJ713257 | Panama | 2005 | Ahumada-Ruiz et al. 2009 |
| FJ713258 | Panama | 2005 | Ahumada-Ruiz et al. 2009 |
| FJ713259 | Panama | 2005 | Ahumada-Ruiz et al. 2009 |
| FJ713260 | Panama | 2005 | Ahumada-Ruiz et al. 2009 |
| FJ713261 | Panama | 2005 | Ahumada-Ruiz et al. 2009 |
| FJ713262 | Panama | 2005 | Ahumada-Ruiz et al. 2009 |
| FJ713263 | Panama | 2005 | Ahumada-Ruiz et al. 2009 |
| FJ713264 | Panama | 2005 | Ahumada-Ruiz et al. 2009 |
| FJ713265 | Panama | 2005 | Ahumada-Ruiz et al. 2009 |
| FJ713266 | Panama | 2005 | Ahumada-Ruiz et al. 2009 |
| FJ713267 | Panama | 2005 | Ahumada-Ruiz et al. 2009 |
| FJ713268 | Panama | 2005 | Ahumada-Ruiz et al. 2009 |
| FJ713269 | Panama | 2005 | Ahumada-Ruiz et al. 2009 |
| FJ713270 | Panama | 2005 | Ahumada-Ruiz et al. 2009 |
| FJ713271 | Panama | 2005 | Ahumada-Ruiz et al. 2009 |
| FJ713272 | Panama | 2005 | Ahumada-Ruiz et al. 2009 |
| FJ713273 | Panama | 2005 | Ahumada-Ruiz et al. 2009 |
| FJ713274 | Panama | 2005 | Ahumada-Ruiz et al. 2009 |
| FJ713275 | Panama | 2005 | Ahumada-Ruiz et al. 2009 |
| FJ713276 | Panama | 2005 | Ahumada-Ruiz et al. 2009 |
| FJ713277 | Panama | 2005 | Ahumada-Ruiz et al. 2009 |
| FJ713278 | Panama | 2005 | Ahumada-Ruiz et al. 2009 |
| FJ713279 | Panama | 2005 | Ahumada-Ruiz et al. 2009 |
| FJ713280 | Panama | 2005 | Ahumada-Ruiz et al. 2009 |
| FJ713281 | Panama | 2005 | Ahumada-Ruiz et al. 2009 |
| FJ713282 | Panama | 2005 | Ahumada-Ruiz et al. 2009 |
| FJ713283 | Panama | 2005 | Ahumada-Ruiz et al. 2009 |
| FJ713284 | Panama | 2005 | Ahumada-Ruiz et al. 2009 |
| FJ713285 | Panama | 2005 | Ahumada-Ruiz et al. 2009 |
| FJ713286 | Panama | 2005 | Ahumada-Ruiz et al. 2009 |
| FJ713287 | Panama | 2005 | Ahumada-Ruiz et al. 2009 |
| FJ713288 | Panama | 2005 | Ahumada-Ruiz et al. 2009 |
| FJ713289 | Panama | 2005 | Ahumada-Ruiz et al. 2009 |
| FJ713290 | Panama | 2005 | Ahumada-Ruiz et al. 2009 |
|  |  |  |  |
|  |  |  |  |
|  |  |  |  |
|  |  |  |  |
|  |  |  |  |
| **The Caribbean** | |  |  |
| AY267265 | Antigua | 2000 | Vaughan et al. 2003 |
| AY267268 | Antigua | 2000 | Vaughan et al. 2003 |
| AY267284 | Antigua | 2000 | Vaughan et al. 2003 |
| AY267286 | Antigua | 2000 | Vaughan et al. 2003 |
| AY267298 | Antigua | 2000 | Vaughan et al. 2003 |
| AY267299 | Antigua | 2000 | Vaughan et al. 2003 |
| AY267300 | Antigua | 2000 | Vaughan et al. 2003 |
| AY267307 | Antigua | 2000 | Vaughan et al. 2003 |
|  |  |  |  |
| DQ518427 | Bahamas | 2004 | Gomez-Carrillo et al. 2006 |
| DQ518428 | Bahamas | 2004 | Gomez-Carrillo et al. 2006 |
| DQ518429 | Bahamas | 2004 | Gomez-Carrillo et al. 2006 |
| DQ518430 | Bahamas | 2004 | Gomez-Carrillo et al. 2006 |
| DQ518431 | Bahamas | 2004 | Gomez-Carrillo et al. 2006 |
| DQ518432 | Bahamas | 2004 | Gomez-Carrillo et al. 2006 |
| DQ518433 | Bahamas | 2004 | Gomez-Carrillo et al. 2006 |
| DQ518434 | Bahamas | 2004 | Gomez-Carrillo et al. 2006 |
| DQ518435 | Bahamas | 2004 | Gomez-Carrillo et al. 2006 |
| DQ518436 | Bahamas | 2004 | Gomez-Carrillo et al. 2006 |
| DQ518437 | Bahamas | 2004 | Gomez-Carrillo et al. 2006 |
| DQ518438 | Bahamas | 2004 | Gomez-Carrillo et al. 2006 |
| DQ518439 | Bahamas | 2004 | Gomez-Carrillo et al. 2006 |
| DQ518440 | Bahamas | 2004 | Gomez-Carrillo et al. 2006 |
|  |  |  |  |
| AY048548/AY116541 | Barbados | 1996 | Gittens et al. 2003 |
| AY048549/AY116542 | Barbados | 1996 | Gittens et al. 2003 |
| AY048550/AY116544 | Barbados | 1996 | Gittens et al. 2003 |
| AY048551/AY116545 | Barbados | 1996 | Gittens et al. 2003 |
| AY048552/AY116546 | Barbados | 1996 | Gittens et al. 2003 |
| AY048553/AY116548 | Barbados | 1996 | Gittens et al. 2003 |
| AY048554/AY116550 | Barbados | 1996 | Gittens et al. 2003 |
| AY048555/AY116553 | Barbados | 1996 | Gittens et al. 2003 |
| AY048556/AY116554 | Barbados | 1996 | Gittens et al. 2003 |
| AY048557/AY116556 | Barbados | 1996 | Gittens et al. 2003 |
| AY048558/AY116557 | Barbados | 1996 | Gittens et al. 2003 |
| AY048561/AY116559 | Barbados | 1996 | Gittens et al. 2003 |
| AY048562/AY116561 | Barbados | 1996 | Gittens et al. 2003 |
| AY048563/AY116562 | Barbados | 1996 | Gittens et al. 2003 |
| AY048564/AY116563 | Barbados | 1996 | Gittens et al. 2003 |
| AY048565/AY116564 | Barbados | 1996 | Gittens et al. 2003 |
| AY048566/AY116567 | Barbados | 1996 | Gittens et al. 2003 |
| AY048567/AY116569 | Barbados | 1996 | Gittens et al. 2003 |
| AY048568/AY116572 | Barbados | 1996 | Gittens et al. 2003 |
| AY048570/AY116574 | Barbados | 1996 | Gittens et al. 2003 |
| AY048571/AY116577 | Barbados | 1996 | Gittens et al. 2003 |
| AY048572/AY116579 | Barbados | 1996 | Gittens et al. 2003 |
| AY048573/AY116581 | Barbados | 1996 | Gittens et al. 2003 |
| AY048574/AY116583 | Barbados | 1996 | Gittens et al. 2003 |
|  |  |  |  |
| AF425382 | Cuba | 1999 | Cuevas et al. 2002 |
| AF425383 | Cuba | 1999 | Cuevas et al. 2002 |
| AF425384 | Cuba | 1999 | Cuevas et al. 2002 |
| AF425386 | Cuba | 1999 | Cuevas et al. 2002 |
| AF425387 | Cuba | 1999 | Cuevas et al. 2002 |
| AF425392 | Cuba | 1999 | Cuevas et al. 2002 |
| AF425393 | Cuba | 1999 | Cuevas et al. 2002 |
| AF425399 | Cuba | 1999 | Cuevas et al. 2002 |
| AF425400 | Cuba | 1999 | Cuevas et al. 2002 |
| AF425402 | Cuba | 1999 | Cuevas et al. 2002 |
| AF425403 | Cuba | 1999 | Cuevas et al. 2002 |
| AF425405 | Cuba | 1999 | Cuevas et al. 2002 |
| AF425406 | Cuba | 1999 | Cuevas et al. 2002 |
| AF425407 | Cuba | 1999 | Cuevas et al. 2002 |
| AF425408 | Cuba | 1999 | Cuevas et al. 2002 |
| AF425413 | Cuba | 1999 | Cuevas et al. 2002 |
| AF425416 | Cuba | 1999 | Cuevas et al. 2002 |
| AF425417 | Cuba | 1999 | Cuevas et al. 2002 |
| AF425418 | Cuba | 1999 | Cuevas et al. 2002 |
| AF425422 | Cuba | 1999 | Cuevas et al. 2002 |
| AF425423 | Cuba | 1999 | Cuevas et al. 2002 |
| AF425424 | Cuba | 1999 | Cuevas et al. 2002 |
| AF425425 | Cuba | 1999 | Cuevas et al. 2002 |
| AF425428 | Cuba | 1999 | Cuevas et al. 2002 |
| AF425429 | Cuba | 1999 | Cuevas et al. 2002 |
| AF425430 | Cuba | 1999 | Cuevas et al. 2002 |
| AF425431 | Cuba | 1999 | Cuevas et al. 2002 |
| AF425433 | Cuba | 1999 | Cuevas et al. 2002 |
| AF425434 | Cuba | 1999 | Cuevas et al. 2002 |
| AF425439 | Cuba | 1999 | Cuevas et al. 2002 |
| AF425440 | Cuba | 1999 | Cuevas et al. 2002 |
| AF425444 | Cuba | 1999 | Cuevas et al. 2002 |
| AF425458 | Cuba | 1999 | Cuevas et al. 2002 |
| AF425460 | Cuba | 1999 | Cuevas et al. 2002 |
| AF425463 | Cuba | 1999 | Cuevas et al. 2002 |
| AF425464 | Cuba | 1999 | Cuevas et al. 2002 |
| AF425465 | Cuba | 1999 | Cuevas et al. 2002 |
| AF425467 | Cuba | 1999 | Cuevas et al. 2002 |
| AF425470 | Cuba | 1999 | Cuevas et al. 2002 |
| AF425471 | Cuba | 1999 | Cuevas et al. 2002 |
| AF425472 | Cuba | 1999 | Cuevas et al. 2002 |
| AF425473 | Cuba | 1999 | Cuevas et al. 2002 |
| AF425474 | Cuba | 1999 | Cuevas et al. 2002 |
| AF425477 | Cuba | 1999 | Cuevas et al. 2002 |
| AF425478 | Cuba | 1999 | Cuevas et al. 2002 |
| AF425479 | Cuba | 1999 | Cuevas et al. 2002 |
| AF425480 | Cuba | 1999 | Cuevas et al. 2002 |
| AF425482 | Cuba | 1999 | Cuevas et al. 2002 |
| AF425483 | Cuba | 1999 | Cuevas et al. 2002 |
| AY586542 | Cuba | 1999 | Cuevas et al. 2002 |
| AY586543 | Cuba | 1999 | Cuevas et al. 2002 |
| DQ112963 | Cuba | 2003 | Perez et al. 2006 |
| DQ112965 | Cuba | 2003 | Perez et al. 2006 |
| DQ112967 | Cuba | 2003 | Perez et al. 2006 |
| DQ112973 | Cuba | 2003 | Perez et al. 2006 |
| DQ112976 | Cuba | 2003 | Perez et al. 2006 |
| DQ112979 | Cuba | 2003 | Perez et al. 2006 |
| DQ112987 | Cuba | 2003 | Perez et al. 2006 |
| DQ112988 | Cuba | 2003 | Perez et al. 2006 |
| DQ112991 | Cuba | 2003 | Perez et al. 2006 |
| DQ112992 | Cuba | 2003 | Perez et al. 2006 |
| DQ112995 | Cuba | 2003 | Perez et al. 2006 |
| DQ112996 | Cuba | 2003 | Perez et al. 2006 |
| DQ112998 | Cuba | 2003 | Perez et al. 2006 |
| DQ113001 | Cuba | 2003 | Perez et al. 2006 |
| DQ113002 | Cuba | 2003 | Perez et al. 2006 |
| DQ113008 | Cuba | 2003 | Perez et al. 2006 |
| DQ113009 | Cuba | 2003 | Perez et al. 2006 |
| DQ113011 | Cuba | 2003 | Perez et al. 2006 |
| DQ113012 | Cuba | 2003 | Perez et al. 2006 |
| DQ113015 | Cuba | 2003 | Perez et al. 2006 |
| DQ113017 | Cuba | 2003 | Perez et al. 2006 |
| DQ113018 | Cuba | 2003 | Perez et al. 2006 |
| DQ113019 | Cuba | 2003 | Perez et al. 2006 |
| DQ113020 | Cuba | 2003 | Perez et al. 2006 |
| DQ113021 | Cuba | 2003 | Perez et al. 2006 |
| DQ113022 | Cuba | 2003 | Perez et al. 2006 |
| DQ113023 | Cuba | 2003 | Perez et al. 2006 |
| DQ113024 | Cuba | 2003 | Perez et al. 2006 |
| DQ113027 | Cuba | 2003 | Perez et al. 2006 |
| DQ113031 | Cuba | 2003 | Perez et al. 2006 |
| DQ113032 | Cuba | 2003 | Perez et al. 2006 |
| DQ113036 | Cuba | 2003 | Perez et al. 2006 |
| DQ113037 | Cuba | 2003 | Perez et al. 2006 |
| DQ113040 | Cuba | 2003 | Perez et al. 2006 |
| DQ113044 | Cuba | 2003 | Perez et al. 2006 |
| DQ113047 | Cuba | 2003 | Perez et al. 2006 |
| DQ113053 | Cuba | 2003 | Perez et al. 2006 |
| DQ113057 | Cuba | 2003 | Perez et al. 2006 |
| DQ113058 | Cuba | 2003 | Perez et al. 2006 |
| DQ113059 | Cuba | 2003 | Perez et al. 2006 |
| DQ113062 | Cuba | 2003 | Perez et al. 2006 |
| DQ113063 | Cuba | 2003 | Perez et al. 2006 |
| DQ113064 | Cuba | 2003 | Perez et al. 2006 |
| DQ113068 | Cuba | 2003 | Perez et al. 2006 |
| DQ113076 | Cuba | 2003 | Perez et al. 2006 |
| DQ113078 | Cuba | 2003 | Perez et al. 2006 |
| DQ113083 | Cuba | 2003 | Perez et al. 2006 |
| DQ113085 | Cuba | 2003 | Perez et al. 2006 |
| DQ113088 | Cuba | 2003 | Perez et al. 2006 |
| DQ113091 | Cuba | 2003 | Perez et al. 2006 |
| DQ113092 | Cuba | 2003 | Perez et al. 2006 |
| DQ113093 | Cuba | 2003 | Perez et al. 2006 |
| DQ113095 | Cuba | 2003 | Perez et al. 2006 |
| DQ113096 | Cuba | 2003 | Perez et al. 2006 |
| DQ113097 | Cuba | 2003 | Perez et al. 2006 |
| DQ113098 | Cuba | 2003 | Perez et al. 2006 |
| DQ113100 | Cuba | 2003 | Perez et al. 2006 |
| DQ113101 | Cuba | 2003 | Perez et al. 2006 |
| DQ113106 | Cuba | 2003 | Perez et al. 2006 |
| DQ113107 | Cuba | 2003 | Perez et al. 2006 |
| DQ113109 | Cuba | 2003 | Perez et al. 2006 |
| DQ113110 | Cuba | 2003 | Perez et al. 2006 |
| DQ113112 | Cuba | 2003 | Perez et al. 2006 |
| DQ113117 | Cuba | 2003 | Perez et al. 2006 |
| DQ113119 | Cuba | 2003 | Perez et al. 2006 |
| DQ113121 | Cuba | 2003 | Perez et al. 2006 |
| DQ113123 | Cuba | 2003 | Perez et al. 2006 |
| DQ113124 | Cuba | 2003 | Perez et al. 2006 |
| DQ113125 | Cuba | 2003 | Perez et al. 2006 |
| DQ113126 | Cuba | 2003 | Perez et al. 2006 |
| DQ113127 | Cuba | 2003 | Perez et al. 2006 |
| DQ113128 | Cuba | 2003 | Perez et al. 2006 |
| DQ113135 | Cuba | 2003 | Perez et al. 2006 |
| DQ113136 | Cuba | 2003 | Perez et al. 2006 |
| DQ113138 | Cuba | 2003 | Perez et al. 2006 |
| DQ113142 | Cuba | 2003 | Perez et al. 2006 |
| DQ113144 | Cuba | 2003 | Perez et al. 2006 |
| DQ113146 | Cuba | 2003 | Perez et al. 2006 |
| DQ113150 | Cuba | 2003 | Perez et al. 2006 |
| DQ113152 | Cuba | 2003 | Perez et al. 2006 |
| DQ113154 | Cuba | 2003 | Perez et al. 2006 |
| DQ113155 | Cuba | 2003 | Perez et al. 2006 |
| DQ113159 | Cuba | 2003 | Perez et al. 2006 |
| DQ113160 | Cuba | 2003 | Perez et al. 2006 |
| DQ113164 | Cuba | 2003 | Perez et al. 2006 |
| DQ113166 | Cuba | 2003 | Perez et al. 2006 |
| DQ113170 | Cuba | 2003 | Perez et al. 2006 |
| DQ113172 | Cuba | 2003 | Perez et al. 2006 |
| DQ113179 | Cuba | 2003 | Perez et al. 2006 |
| DQ113191 | Cuba | 2003 | Perez et al. 2006 |
| DQ113192 | Cuba | 2003 | Perez et al. 2006 |
| DQ113193 | Cuba | 2003 | Perez et al. 2006 |
| DQ113195 | Cuba | 2003 | Perez et al. 2006 |
| DQ113197 | Cuba | 2003 | Perez et al. 2006 |
| DQ113201 | Cuba | 2003 | Perez et al. 2006 |
| DQ113202 | Cuba | 2003 | Perez et al. 2006 |
| DQ113203 | Cuba | 2003 | Perez et al. 2006 |
| DQ113207 | Cuba | 2003 | Perez et al. 2006 |
| DQ113208 | Cuba | 2003 | Perez et al. 2006 |
| DQ113210 | Cuba | 2003 | Perez et al. 2006 |
| DQ113214 | Cuba | 2003 | Perez et al. 2006 |
| DQ113215 | Cuba | 2003 | Perez et al. 2006 |
| DQ113220 | Cuba | 2003 | Perez et al. 2006 |
| DQ113221 | Cuba | 2003 | Perez et al. 2006 |
| DQ113222 | Cuba | 2003 | Perez et al. 2006 |
| DQ113225 | Cuba | 2003 | Perez et al. 2006 |
| DQ113227 | Cuba | 2003 | Perez et al. 2006 |
| DQ113230 | Cuba | 2003 | Perez et al. 2006 |
| DQ113235 | Cuba | 2003 | Perez et al. 2006 |
| DQ113236 | Cuba | 2003 | Perez et al. 2006 |
| DQ113237 | Cuba | 2003 | Perez et al. 2006 |
| DQ113239 | Cuba | 2003 | Perez et al. 2006 |
| DQ113240 | Cuba | 2003 | Perez et al. 2006 |
| DQ113241 | Cuba | 2003 | Perez et al. 2006 |
| DQ113244 | Cuba | 2003 | Perez et al. 2006 |
| DQ113245 | Cuba | 2003 | Perez et al. 2006 |
| DQ113246 | Cuba | 2003 | Perez et al. 2006 |
| DQ113247 | Cuba | 2003 | Perez et al. 2006 |
| DQ113249 | Cuba | 2003 | Perez et al. 2006 |
| DQ113250 | Cuba | 2003 | Perez et al. 2006 |
| DQ113251 | Cuba | 2003 | Perez et al. 2006 |
| DQ113252 | Cuba | 2003 | Perez et al. 2006 |
| DQ113253 | Cuba | 2003 | Perez et al. 2006 |
| DQ113255 | Cuba | 2003 | Perez et al. 2006 |
| DQ113256 | Cuba | 2003 | Perez et al. 2006 |
| DQ113259 | Cuba | 2003 | Perez et al. 2006 |
| DQ113261 | Cuba | 2003 | Perez et al. 2006 |
| DQ113262 | Cuba | 2003 | Perez et al. 2006 |
| DQ113265 | Cuba | 2003 | Perez et al. 2006 |
| DQ113266 | Cuba | 2003 | Perez et al. 2006 |
| DQ113275 | Cuba | 2003 | Perez et al. 2006 |
| DQ113276 | Cuba | 2003 | Perez et al. 2006 |
| DQ113277 | Cuba | 2003 | Perez et al. 2006 |
| DQ113279 | Cuba | 2003 | Perez et al. 2006 |
| DQ113280 | Cuba | 2003 | Perez et al. 2006 |
| DQ113281 | Cuba | 2003 | Perez et al. 2006 |
| DQ113283 | Cuba | 2003 | Perez et al. 2006 |
| DQ113284 | Cuba | 2003 | Perez et al. 2006 |
| DQ113285 | Cuba | 2003 | Perez et al. 2006 |
| DQ113286 | Cuba | 2003 | Perez et al. 2006 |
| DQ113287 | Cuba | 2003 | Perez et al. 2006 |
| DQ113290 | Cuba | 2003 | Perez et al. 2006 |
| DQ113301 | Cuba | 2003 | Perez et al. 2006 |
| DQ113303 | Cuba | 2003 | Perez et al. 2006 |
| DQ113305 | Cuba | 2003 | Perez et al. 2006 |
| DQ113309 | Cuba | 2003 | Perez et al. 2006 |
| DQ113314 | Cuba | 2003 | Perez et al. 2006 |
| DQ113320 | Cuba | 2003 | Perez et al. 2006 |
| DQ113322 | Cuba | 2003 | Perez et al. 2006 |
| DQ113324 | Cuba | 2003 | Perez et al. 2006 |
| DQ113325 | Cuba | 2003 | Perez et al. 2006 |
| DQ113328 | Cuba | 2003 | Perez et al. 2006 |
| DQ113330 | Cuba | 2003 | Perez et al. 2006 |
| DQ113331 | Cuba | 2003 | Perez et al. 2006 |
| DQ113333 | Cuba | 2003 | Perez et al. 2006 |
| DQ113334 | Cuba | 2003 | Perez et al. 2006 |
| DQ113335 | Cuba | 2003 | Perez et al. 2006 |
| DQ113337 | Cuba | 2003 | Perez et al. 2006 |
| DQ113342 | Cuba | 2003 | Perez et al. 2006 |
| DQ113346 | Cuba | 2003 | Perez et al. 2006 |
| DQ113349 | Cuba | 2003 | Perez et al. 2006 |
| DQ113351 | Cuba | 2003 | Perez et al. 2006 |
| DQ113352 | Cuba | 2003 | Perez et al. 2006 |
| DQ113353 | Cuba | 2003 | Perez et al. 2006 |
| DQ113355 | Cuba | 2003 | Perez et al. 2006 |
| DQ113358 | Cuba | 2003 | Perez et al. 2006 |
| DQ113361 | Cuba | 2003 | Perez et al. 2006 |
| DQ113366 | Cuba | 2003 | Perez et al. 2006 |
| DQ113367 | Cuba | 2003 | Perez et al. 2006 |
| DQ113368 | Cuba | 2003 | Perez et al. 2006 |
| DQ113371 | Cuba | 2003 | Perez et al. 2006 |
| DQ113374 | Cuba | 2003 | Perez et al. 2006 |
| DQ113376 | Cuba | 2003 | Perez et al. 2006 |
| DQ113377 | Cuba | 2003 | Perez et al. 2006 |
| DQ113380 | Cuba | 2003 | Perez et al. 2006 |
| DQ320459 | Cuba | 1992 | Resik et al. 2007 |
| DQ320466 | Cuba | 2000 | Resik et al. 2007 |
| HQ108355 | Cuba | 2006 | Perez et al. 2011 |
| HQ108358 | Cuba | 2008 | Perez et al. 2011 |
| HQ108359 | Cuba | 2006 | Perez et al. 2011 |
| HQ108363 | Cuba | 2005 | Perez et al. 2011 |
| HQ108365 | Cuba | 2006 | Perez et al. 2011 |
| HQ655338 | Cuba | 2009 | Machado et al. 2011 |
| HQ655340 | Cuba | 2009 | Machado et al. 2011 |
| HQ655341 | Cuba | 2009 | Machado et al. 2011 |
| HQ655345 | Cuba | 2009 | Machado et al. 2011 |
| HQ655350 | Cuba | 2009 | Machado et al. 2011 |
| HQ655351 | Cuba | 2009 | Machado et al. 2011 |
| HQ655352 | Cuba | 2009 | Machado et al. 2011 |
| HQ655354 | Cuba | 2009 | Machado et al. 2011 |
| HQ655355 | Cuba | 2009 | Machado et al. 2011 |
| HQ655356 | Cuba | 2009 | Machado et al. 2011 |
| HQ655360 | Cuba | 2009 | Machado et al. 2011 |
| HQ655363 | Cuba | 2009 | Machado et al. 2011 |
| HQ655366 | Cuba | 2009 | Machado et al. 2011 |
| HQ655368 | Cuba | 2009 | Machado et al. 2011 |
| HQ655372 | Cuba | 2009 | Machado et al. 2011 |
| HQ655375 | Cuba | 2009 | Machado et al. 2011 |
| HQ655377 | Cuba | 2009 | Machado et al. 2011 |
| HQ655379 | Cuba | 2010 | Machado et al. 2011 |
| HQ655380 | Cuba | 2010 | Machado et al. 2011 |
| HQ655381 | Cuba | 2010 | Machado et al. 2011 |
| HQ655383 | Cuba | 2010 | Machado et al. 2011 |
| HQ655387 | Cuba | 2010 | Machado et al. 2011 |
| HQ655390 | Cuba | 2010 | Machado et al. 2011 |
| HQ655393 | Cuba | 2010 | Machado et al. 2011 |
| HQ655394 | Cuba | 2010 | Machado et al. 2011 |
| HQ655396 | Cuba | 2010 | Machado et al. 2011 |
| HQ655399 | Cuba | 2010 | Machado et al. 2011 |
| HQ655401 | Cuba | 2010 | Machado et al. 2011 |
| HQ655409 | Cuba | 2010 | Machado et al. 2011 |
| JN000005 | Cuba | 2010 | Machado et al. 2011 |
| JN000006 | Cuba | 2010 | Machado et al. 2011 |
| JN000007 | Cuba | 2010 | Machado et al. 2011 |
| JN000008 | Cuba | 2010 | Machado et al. 2011 |
| JN000009 | Cuba | 2010 | Machado et al. 2011 |
| JN000010 | Cuba | 2010 | Machado et al. 2011 |
| JN000011 | Cuba | 2010 | Machado et al. 2011 |
| JN000016 | Cuba | 2010 | Machado et al. 2011 |
| JN000025 | Cuba | 2010 | Machado et al. 2011 |
| JN000033 | Cuba | 2010 | Machado et al. 2011 |
| JN000037 | Cuba | 2010 | Machado et al. 2011 |
| JN000038 | Cuba | 2010 | Machado et al. 2011 |
| JN000041 | Cuba | 2010 | Machado et al. 2011 |
| JN000048 | Cuba | 2010 | Machado et al. 2011 |
| JN000050 | Cuba | 2010 | Machado et al. 2011 |
| JN000061 | Cuba | 2010 | Machado et al. 2011 |
|  |  |  |  |
| AY267295 | Dominica | 2000 | Vaughan et al. 2003 |
| AY267296 | Dominica | 2000 | Vaughan et al. 2003 |
| AY267297 | Dominica | 2000 | Vaughan et al. 2003 |
| AY267298 | Dominica | 2000 | Vaughan et al. 2003 |
|  |  |  |  |
| EU839597 | Dominican Rep. | 2005 | Nadai et al. 2009 |
| EU839598 | Dominican Rep. | 2005 | Nadai et al. 2009 |
| EU839596 | Dominican Rep. | 2005 | Nadai et al. 2009 |
| EU439763 | Dominican Rep. | 2003 | Nadai et al. 2009 |
| EU439709 | Dominican Rep. | 2005 | Nadai et al. 2009 |
| EU439711 | Dominican Rep. | 2005 | Nadai et al. 2009 |
| EU439716 | Dominican Rep. | 2005 | Nadai et al. 2009 |
| EU439761 | Dominican Rep. | 2003 | Nadai et al. 2009 |
| EU439762 | Dominican Rep. | 2003 | Nadai et al. 2009 |
| EU439760 | Dominican Rep. | 2002 | Nadai et al. 2009 |
| EU439764 | Dominican Rep. | 2003 | Nadai et al. 2009 |
| EU439765 | Dominican Rep. | 2003 | Nadai et al. 2009 |
| EU439766 | Dominican Rep. | 2003 | Nadai et al. 2009 |
| EU439767 | Dominican Rep. | 2003 | Nadai et al. 2009 |
| EU439768 | Dominican Rep. | 2003 | Nadai et al. 2009 |
| EU439769 | Dominican Rep. | 2003 | Nadai et al. 2009 |
| EU439770 | Dominican Rep. | 2003 | Nadai et al. 2009 |
| EU439771 | Dominican Rep. | 2004 | Nadai et al. 2009 |
| EU439772 | Dominican Rep. | 2003 | Nadai et al. 2009 |
| EU439715 | Dominican Rep. | 2005 | Nadai et al. 2009 |
|  |  |  |  |
| AY267294 | Grenada | 2000 | Vaughan et al. 2003 |
| AY267303 | Grenada | 2000 | Vaughan et al. 2003 |
| AY267304 | Grenada | 2000 | Vaughan et al. 2003 |
| AY267323 | Grenada | 2000 | Vaughan et al. 2003 |
|  |  |  |  |
| EU839604 | Haiti | 2005 | Nadai et al. 2009 |
| EU839602 | Haiti | 2005 | Nadai et al. 2009 |
| EU839603 | Haiti | 2005 | Nadai et al. 2009 |
| EU839601 | Haiti | 2005 | Nadai et al. 2009 |
| EU839600 | Haiti | 2005 | Nadai et al. 2009 |
| EU439722 | Haiti | 2005 | Nadai et al. 2009 |
| EU439719 | Haiti | 2005 | Nadai et al. 2009 |
| EU439718 | Haiti | 2004 | Nadai et al. 2009 |
| EU439717 | Haiti | 2005 | Nadai et al. 2009 |
| EU439724 | Haiti | 2005 | Nadai et al. 2009 |
| EU439725 | Haiti | 2005 | Nadai et al. 2009 |
| EU439726 | Haiti | 2005 | Nadai et al. 2009 |
| EU439727 | Haiti | 2005 | Nadai et al. 2009 |
| EU439728 | Haiti | 2004 | Nadai et al. 2009 |
| EU439729 | Haiti | 2005 | Nadai et al. 2009 |
| EU439713 | Haiti | 2005 | Nadai et al. 2009 |
| EU439714 | Haiti | 2005 | Nadai et al. 2009 |
| EU439721 | Haiti | 2005 | Nadai et al. 2009 |
| EU439712 | Haiti | 2005 | Nadai et al. 2009 |
| EU439720 | Haiti | 2005 | Nadai et al. 2009 |
| EU439723 | Haiti | 2005 | Nadai et al. 2009 |
|  |  |  |  |
| EU439710 | Jamaica | 2005 | Nadai et al. 2009 |
| EU439773 | Jamaica | 2001 | Nadai et al. 2009 |
| EU439774 | Jamaica | 2001 | Nadai et al. 2009 |
| EU839605 | Jamaica | 2005 | Nadai et al. 2009 |
| GU437877 | Jamaica | 2009 | Hamilton et al. 2012 |
| GU437878 | Jamaica | 2009 | Hamilton et al. 2012 |
| GU437879 | Jamaica | 2009 | Hamilton et al. 2012 |
| GU437880 | Jamaica | 2009 | Hamilton et al. 2012 |
| GU437881 | Jamaica | 2008 | Hamilton et al. 2012 |
| GU437882 | Jamaica | 2009 | Hamilton et al. 2012 |
| GU437883 | Jamaica | 2009 | Hamilton et al. 2012 |
| GU437884 | Jamaica | 2009 | Hamilton et al. 2012 |
| GU437885 | Jamaica | 2009 | Hamilton et al. 2012 |
| GU437886 | Jamaica | 2009 | Hamilton et al. 2012 |
| GU437887 | Jamaica | 2009 | Hamilton et al. 2012 |
| GU437888 | Jamaica | 2009 | Hamilton et al. 2012 |
| GU437889 | Jamaica | 2009 | Hamilton et al. 2012 |
| GU437890 | Jamaica | 2009 | Hamilton et al. 2012 |
| GU437891 | Jamaica | 2009 | Hamilton et al. 2012 |
| GU437892 | Jamaica | 2009 | Hamilton et al. 2012 |
| GU437893 | Jamaica | 2009 | Hamilton et al. 2012 |
| GU437894 | Jamaica | 2009 | Hamilton et al. 2012 |
| GU437895 | Jamaica | 2009 | Hamilton et al. 2012 |
| GU437896 | Jamaica | 2009 | Hamilton et al. 2012 |
| GU437897 | Jamaica | 2009 | Hamilton et al. 2012 |
| GU437898 | Jamaica | 2009 | Hamilton et al. 2012 |
| GU437899 | Jamaica | 2009 | Hamilton et al. 2012 |
| GU437900 | Jamaica | 2009 | Hamilton et al. 2012 |
| GU437901 | Jamaica | 2009 | Hamilton et al. 2012 |
| GU437902 | Jamaica | 2009 | Hamilton et al. 2012 |
| GU437903 | Jamaica | 2009 | Hamilton et al. 2012 |
| GU437904 | Jamaica | 2009 | Hamilton et al. 2012 |
| GU437905 | Jamaica | 2009 | Hamilton et al. 2012 |
| GU437906 | Jamaica | 2009 | Hamilton et al. 2012 |
| GU437907 | Jamaica | 2009 | Hamilton et al. 2012 |
| GU437908 | Jamaica | 2009 | Hamilton et al. 2012 |
| GU437909 | Jamaica | 2009 | Hamilton et al. 2012 |
| GU437910 | Jamaica | 2009 | Hamilton et al. 2012 |
| GU437911 | Jamaica | 2009 | Hamilton et al. 2012 |
| GU437912 | Jamaica | 2009 | Hamilton et al. 2012 |
| GU437913 | Jamaica | 2009 | Hamilton et al. 2012 |
| GU972719 | Jamaica | 2009 | Hamilton et al. 2012 |
| GU972720 | Jamaica | 2009 | Hamilton et al. 2012 |
| GU972721 | Jamaica | 2009 | Hamilton et al. 2012 |
| GU972722 | Jamaica | 2009 | Hamilton et al. 2012 |
| GU972723 | Jamaica | 2009 | Hamilton et al. 2012 |
| GU972724 | Jamaica | 2009 | Hamilton et al. 2012 |
| GU972725 | Jamaica | 2009 | Hamilton et al. 2012 |
| GU972726 | Jamaica | 2009 | Hamilton et al. 2012 |
| GU972727 | Jamaica | 2009 | Hamilton et al. 2012 |
| GU972728 | Jamaica | 2009 | Hamilton et al. 2012 |
| GU972729 | Jamaica | 2009 | Hamilton et al. 2012 |
| GU972730 | Jamaica | 2009 | Hamilton et al. 2012 |
| GU972731 | Jamaica | 2009 | Hamilton et al. 2012 |
| GU972732 | Jamaica | 2009 | Hamilton et al. 2012 |
| GU972733 | Jamaica | 2009 | Hamilton et al. 2012 |
| GU972734 | Jamaica | 2009 | Hamilton et al. 2012 |
| GU972735 | Jamaica | 2009 | Hamilton et al. 2012 |
| GU972736 | Jamaica | 2009 | Hamilton et al. 2012 |
| GU972737 | Jamaica | 2009 | Hamilton et al. 2012 |
| GU972738 | Jamaica | 2009 | Hamilton et al. 2012 |
| GU972739 | Jamaica | 2009 | Hamilton et al. 2012 |
| GU972740 | Jamaica | 2009 | Hamilton et al. 2012 |
| GU972741 | Jamaica | 2009 | Hamilton et al. 2012 |
| GU972742 | Jamaica | 2009 | Hamilton et al. 2012 |
| GU972743 | Jamaica | 2009 | Hamilton et al. 2012 |
| GU972744 | Jamaica | 2009 | Hamilton et al. 2012 |
| GU972745 | Jamaica | 2009 | Hamilton et al. 2012 |
| GU972746 | Jamaica | 2009 | Hamilton et al. 2012 |
| GU972747 | Jamaica | 2009 | Hamilton et al. 2012 |
| GU972748 | Jamaica | 2009 | Hamilton et al. 2012 |
| GU972749 | Jamaica | 2009 | Hamilton et al. 2012 |
| GU972750 | Jamaica | 2009 | Hamilton et al. 2012 |
| GU972751 | Jamaica | 2009 | Hamilton et al. 2012 |
| GU972752 | Jamaica | 2009 | Hamilton et al. 2012 |
| GU972753 | Jamaica | 2009 | Hamilton et al. 2012 |
| GU972754 | Jamaica | 2009 | Hamilton et al. 2012 |
| GU972755 | Jamaica | 2009 | Hamilton et al. 2012 |
| GU972756 | Jamaica | 2009 | Hamilton et al. 2012 |
| GU972757 | Jamaica | 2009 | Hamilton et al. 2012 |
| GU972758 | Jamaica | 2009 | Hamilton et al. 2012 |
| GU972759 | Jamaica | 2009 | Hamilton et al. 2012 |
| GU972760 | Jamaica | 2009 | Hamilton et al. 2012 |
| GU972761 | Jamaica | 2009 | Hamilton et al. 2012 |
| GU972762 | Jamaica | 2009 | Hamilton et al. 2012 |
| GU972763 | Jamaica | 2009 | Hamilton et al. 2012 |
| GU972764 | Jamaica | 2009 | Hamilton et al. 2012 |
| GU972765 | Jamaica | 2009 | Hamilton et al. 2012 |
| GU972766 | Jamaica | 2009 | Hamilton et al. 2012 |
| GU972767 | Jamaica | 2009 | Hamilton et al. 2012 |
| GU972768 | Jamaica | 2009 | Hamilton et al. 2012 |
| GU972769 | Jamaica | 2009 | Hamilton et al. 2012 |
| GU972770 | Jamaica | 2009 | Hamilton et al. 2012 |
| GU972771 | Jamaica | 2009 | Hamilton et al. 2012 |
| GU972772 | Jamaica | 2009 | Hamilton et al. 2012 |
| GU972773 | Jamaica | 2009 | Hamilton et al. 2012 |
| GU972774 | Jamaica | 2009 | Hamilton et al. 2012 |
| GU972775 | Jamaica | 2009 | Hamilton et al. 2012 |
| GU972776 | Jamaica | 2009 | Hamilton et al. 2012 |
| GU972777 | Jamaica | 2009 | Hamilton et al. 2012 |
| GU972778 | Jamaica | 2009 | Hamilton et al. 2012 |
| GU972779 | Jamaica | 2009 | Hamilton et al. 2012 |
| GU972780 | Jamaica | 2009 | Hamilton et al. 2012 |
| GU972781 | Jamaica | 2009 | Hamilton et al. 2012 |
| GU972782 | Jamaica | 2009 | Hamilton et al. 2012 |
| GU972783 | Jamaica | 2009 | Hamilton et al. 2012 |
| HM030559 | Jamaica | 2009 | Roye et al. 2011 |
| HM030560 | Jamaica | 2009 | Roye et al. 2011 |
| HM030561 | Jamaica | 2009 | Roye et al. 2011 |
| HM030562 | Jamaica | 2009 | Roye et al. 2011 |
| HM030563 | Jamaica | 2009 | Roye et al. 2011 |
| HM030564 | Jamaica | 2009 | Roye et al. 2011 |
| HM030565 | Jamaica | 2009 | Roye et al. 2011 |
|  |  |  |  |
| AF373732 | Puerto Rico | 1996 | Machado et al. 2002 |
| AF373733 | Puerto Rico | 1996 | Machado et al. 2002 |
| AF373736 | Puerto Rico | 1996 | Machado et al. 2002 |
| AF373737 | Puerto Rico | 1996 | Machado et al. 2002 |
| AF373738 | Puerto Rico | 1996 | Machado et al. 2002 |
| AY334871 | Puerto Rico | 2001 | Noel et al. 2003 |
| AY334872 | Puerto Rico | 2001 | Noel et al. 2003 |
| AY334873 | Puerto Rico | 2001 | Noel et al. 2003 |
| AY334874 | Puerto Rico | 2001 | Noel et al. 2003 |
| AY334875 | Puerto Rico | 2001 | Noel et al. 2003 |
| AY334876 | Puerto Rico | 2001 | Noel et al. 2003 |
| AY334877 | Puerto Rico | 2001 | Noel et al. 2003 |
| AY334878 | Puerto Rico | 2001 | Noel et al. 2003 |
| AY334879 | Puerto Rico | 2001 | Noel et al. 2003 |
| AY334880 | Puerto Rico | 2001 | Noel et al. 2003 |
| AY334881 | Puerto Rico | 2001 | Noel et al. 2003 |
| AY334882 | Puerto Rico | 2001 | Noel et al. 2003 |
| AY334883 | Puerto Rico | 2001 | Noel et al. 2003 |
| AY334886 | Puerto Rico | 2001 | Noel et al. 2003 |
| AY334887 | Puerto Rico | 2001 | Noel et al. 2003 |
| AY334888 | Puerto Rico | 2001 | Noel et al. 2003 |
| AY334889 | Puerto Rico | 2001 | Noel et al. 2003 |
| AY334890 | Puerto Rico | 2001 | Noel et al. 2003 |
| AY334891 | Puerto Rico | 2001 | Noel et al. 2003 |
| AY334892 | Puerto Rico | 2001 | Noel et al. 2003 |
| AY334893 | Puerto Rico | 2001 | Noel et al. 2003 |
| AY334894 | Puerto Rico | 2001 | Noel et al. 2003 |
| AY334895 | Puerto Rico | 2001 | Noel et al. 2003 |
| AY334896 | Puerto Rico | 2001 | Noel et al. 2003 |
| AY334897 | Puerto Rico | 2001 | Noel et al. 2003 |
| AY334898 | Puerto Rico | 2001 | Noel et al. 2003 |
| AY334899 | Puerto Rico | 2001 | Noel et al. 2003 |
| AY334900 | Puerto Rico | 2001 | Noel et al. 2003 |
| AY334901 | Puerto Rico | 2001 | Noel et al. 2003 |
| AY334902 | Puerto Rico | 2001 | Noel et al. 2003 |
| AY334903 | Puerto Rico | 2001 | Noel et al. 2003 |
| AY334904 | Puerto Rico | 2001 | Noel et al. 2003 |
| AY334905 | Puerto Rico | 2001 | Noel et al. 2003 |
| AY334906 | Puerto Rico | 2001 | Noel et al. 2003 |
| AY334907 | Puerto Rico | 2001 | Noel et al. 2003 |
| AY334909 | Puerto Rico | 2001 | Noel et al. 2003 |
| AY334910 | Puerto Rico | 2001 | Noel et al. 2003 |
| AY334911 | Puerto Rico | 2001 | Noel et al. 2003 |
| AY334912 | Puerto Rico | 2001 | Noel et al. 2003 |
| AY334913 | Puerto Rico | 2001 | Noel et al. 2003 |
| AY334914 | Puerto Rico | 2001 | Noel et al. 2003 |
| AY334915 | Puerto Rico | 2001 | Noel et al. 2003 |
| AY334916 | Puerto Rico | 2001 | Noel et al. 2003 |
| AY334917 | Puerto Rico | 2001 | Noel et al. 2003 |
| AY334918 | Puerto Rico | 2001 | Noel et al. 2003 |
| AY334919 | Puerto Rico | 2001 | Noel et al. 2003 |
| AY334920 | Puerto Rico | 2001 | Noel et al. 2003 |
| AY334921 | Puerto Rico | 2001 | Noel et al. 2003 |
| AY334922 | Puerto Rico | 2001 | Noel et al. 2003 |
| AY334923 | Puerto Rico | 2001 | Noel et al. 2003 |
| AY334925 | Puerto Rico | 2001 | Noel et al. 2003 |
| AY334926 | Puerto Rico | 2001 | Noel et al. 2003 |
| AY334927 | Puerto Rico | 2001 | Noel et al. 2003 |
| AY334928 | Puerto Rico | 2001 | Noel et al. 2003 |
| AY334929 | Puerto Rico | 2001 | Noel et al. 2003 |
| AY334930 | Puerto Rico | 2001 | Noel et al. 2003 |
| AY334932 | Puerto Rico | 2001 | Noel et al. 2003 |
| AY334933 | Puerto Rico | 2001 | Noel et al. 2003 |
| AY334934 | Puerto Rico | 2001 | Noel et al. 2003 |
| AY334935 | Puerto Rico | 2001 | Noel et al. 2003 |
| AY334936 | Puerto Rico | 2001 | Noel et al. 2003 |
| AY334937 | Puerto Rico | 2001 | Noel et al. 2003 |
| AY334938 | Puerto Rico | 2001 | Noel et al. 2003 |
| AY334939 | Puerto Rico | 2001 | Noel et al. 2003 |
| AY334940 | Puerto Rico | 2001 | Noel et al. 2003 |
| AY334941 | Puerto Rico | 2001 | Noel et al. 2003 |
| AY334942 | Puerto Rico | 2001 | Noel et al. 2003 |
| AY334943 | Puerto Rico | 2001 | Noel et al. 2003 |
| AY334944 | Puerto Rico | 2001 | Noel et al. 2003 |
| AY334945 | Puerto Rico | 2001 | Noel et al. 2003 |
| AY334946 | Puerto Rico | 2001 | Noel et al. 2003 |
| AY334947 | Puerto Rico | 2001 | Noel et al. 2003 |
| AY334948 | Puerto Rico | 2001 | Noel et al. 2003 |
| AY334949 | Puerto Rico | 2001 | Noel et al. 2003 |
| AY334950 | Puerto Rico | 2001 | Noel et al. 2003 |
| AY334951 | Puerto Rico | 2001 | Noel et al. 2003 |
| AY334952 | Puerto Rico | 2001 | Noel et al. 2003 |
| AY334953 | Puerto Rico | 2001 | Noel et al. 2003 |
| AY334954 | Puerto Rico | 2001 | Noel et al. 2003 |
| AY334955 | Puerto Rico | 2001 | Noel et al. 2003 |
| AY334956 | Puerto Rico | 2001 | Noel et al. 2003 |
| AY334957 | Puerto Rico | 2001 | Noel et al. 2003 |
| AY334958 | Puerto Rico | 2001 | Noel et al. 2003 |
| AY334959 | Puerto Rico | 2001 | Noel et al. 2003 |
| AY334960 | Puerto Rico | 2001 | Noel et al. 2003 |
| AY334961 | Puerto Rico | 2001 | Noel et al. 2003 |
| AY334962 | Puerto Rico | 2001 | Noel et al. 2003 |
| AY334963 | Puerto Rico | 2001 | Noel et al. 2003 |
| AY334964 | Puerto Rico | 2001 | Noel et al. 2003 |
| AY334965 | Puerto Rico | 2001 | Noel et al. 2003 |
| AY334966 | Puerto Rico | 2001 | Noel et al. 2003 |
| AY334967 | Puerto Rico | 2001 | Noel et al. 2003 |
| AY334968 | Puerto Rico | 2001 | Noel et al. 2003 |
| AY334969 | Puerto Rico | 2001 | Noel et al. 2003 |
| AY334970 | Puerto Rico | 2001 | Noel et al. 2003 |
| AY334971 | Puerto Rico | 2001 | Noel et al. 2003 |
| AY334973 | Puerto Rico | 2001 | Noel et al. 2003 |
| AY334974 | Puerto Rico | 2001 | Noel et al. 2003 |
| AY334975 | Puerto Rico | 2001 | Noel et al. 2003 |
| AY334976 | Puerto Rico | 2001 | Noel et al. 2003 |
| AY334977 | Puerto Rico | 2001 | Noel et al. 2003 |
| AY334978 | Puerto Rico | 2001 | Noel et al. 2003 |
| AY334979 | Puerto Rico | 2001 | Noel et al. 2003 |
| AY334980 | Puerto Rico | 2001 | Noel et al. 2003 |
| AY334981 | Puerto Rico | 2001 | Noel et al. 2003 |
| AY334982 | Puerto Rico | 2001 | Noel et al. 2003 |
| AY334983 | Puerto Rico | 2001 | Noel et al. 2003 |
| AY334984 | Puerto Rico | 2001 | Noel et al. 2003 |
| AY334985 | Puerto Rico | 2001 | Noel et al. 2003 |
| AY334986 | Puerto Rico | 2001 | Noel et al. 2003 |
| AY334987 | Puerto Rico | 2001 | Noel et al. 2003 |
| AY334988 | Puerto Rico | 2001 | Noel et al. 2003 |
| AY334989 | Puerto Rico | 2001 | Noel et al. 2003 |
| AY334990 | Puerto Rico | 2001 | Noel et al. 2003 |
| AY334991 | Puerto Rico | 2001 | Noel et al. 2003 |
| AY334992 | Puerto Rico | 2001 | Noel et al. 2003 |
| AY334993 | Puerto Rico | 2001 | Noel et al. 2003 |
| AY334996 | Puerto Rico | 2001 | Noel et al. 2003 |
| AY334997 | Puerto Rico | 2001 | Noel et al. 2003 |
| AY334998 | Puerto Rico | 2001 | Noel et al. 2003 |
| AY334999 | Puerto Rico | 2001 | Noel et al. 2003 |
| AY335000 | Puerto Rico | 2001 | Noel et al. 2003 |
| AY335001 | Puerto Rico | 2001 | Noel et al. 2003 |
| AY335002 | Puerto Rico | 2001 | Noel et al. 2003 |
| AY335003 | Puerto Rico | 2001 | Noel et al. 2003 |
| AY335004 | Puerto Rico | 2001 | Noel et al. 2003 |
| AY335005 | Puerto Rico | 2001 | Noel et al. 2003 |
| AY335006 | Puerto Rico | 2001 | Noel et al. 2003 |
| AY335007 | Puerto Rico | 2001 | Noel et al. 2003 |
| AY335008 | Puerto Rico | 2001 | Noel et al. 2003 |
| AY335009 | Puerto Rico | 2001 | Noel et al. 2003 |
| AY335010 | Puerto Rico | 2001 | Noel et al. 2003 |
| AY335011 | Puerto Rico | 2001 | Noel et al. 2003 |
| AY335012 | Puerto Rico | 2001 | Noel et al. 2003 |
| AY335013 | Puerto Rico | 2001 | Noel et al. 2003 |
| AY335014 | Puerto Rico | 2001 | Noel et al. 2003 |
| AY335015 | Puerto Rico | 2001 | Noel et al. 2003 |
| AY335016 | Puerto Rico | 2001 | Noel et al. 2003 |
| AY335017 | Puerto Rico | 2001 | Noel et al. 2003 |
| AY335018 | Puerto Rico | 2001 | Noel et al. 2003 |
| AY335019 | Puerto Rico | 2001 | Noel et al. 2003 |
| AY335020 | Puerto Rico | 2001 | Noel et al. 2003 |
| AY335021 | Puerto Rico | 2001 | Noel et al. 2003 |
| AY335023 | Puerto Rico | 2001 | Noel et al. 2003 |
| AY335024 | Puerto Rico | 2001 | Noel et al. 2003 |
| AY335025 | Puerto Rico | 2001 | Noel et al. 2003 |
| AY335026 | Puerto Rico | 2001 | Noel et al. 2003 |
| AY335027 | Puerto Rico | 2001 | Noel et al. 2003 |
| AY335028 | Puerto Rico | 2001 | Noel et al. 2003 |
| AY335029 | Puerto Rico | 2001 | Noel et al. 2003 |
| AY335030 | Puerto Rico | 2001 | Noel et al. 2003 |
| AY335031 | Puerto Rico | 2001 | Noel et al. 2003 |
| AY335032 | Puerto Rico | 2001 | Noel et al. 2003 |
| AY335033 | Puerto Rico | 2001 | Noel et al. 2003 |
| AY335034 | Puerto Rico | 2001 | Noel et al. 2003 |
| AY335035 | Puerto Rico | 2001 | Noel et al. 2003 |
| AY335037 | Puerto Rico | 2001 | Noel et al. 2003 |
| AY335038 | Puerto Rico | 2001 | Noel et al. 2003 |
| AY335039 | Puerto Rico | 2001 | Noel et al. 2003 |
| AY335040 | Puerto Rico | 2001 | Noel et al. 2003 |
| AY335041 | Puerto Rico | 2001 | Noel et al. 2003 |
| AY335042 | Puerto Rico | 2001 | Noel et al. 2003 |
| AY335043 | Puerto Rico | 2001 | Noel et al. 2003 |
| AY335045 | Puerto Rico | 2001 | Noel et al. 2003 |
| AY335046 | Puerto Rico | 2001 | Noel et al. 2003 |
| AY335049 | Puerto Rico | 2001 | Noel et al. 2003 |
| AY335050 | Puerto Rico | 2001 | Noel et al. 2003 |
| AY335052 | Puerto Rico | 2001 | Noel et al. 2003 |
| AY335053 | Puerto Rico | 2001 | Noel et al. 2003 |
| AY335054 | Puerto Rico | 2001 | Noel et al. 2003 |
| AY335055 | Puerto Rico | 2001 | Noel et al. 2003 |
| AY335056 | Puerto Rico | 2001 | Noel et al. 2003 |
| AY335057 | Puerto Rico | 2001 | Noel et al. 2003 |
| AY335058 | Puerto Rico | 2001 | Noel et al. 2003 |
| AY335059 | Puerto Rico | 2001 | Noel et al. 2003 |
| AY335060 | Puerto Rico | 2001 | Noel et al. 2003 |
| AY335061 | Puerto Rico | 2001 | Noel et al. 2003 |
| AY335062 | Puerto Rico | 2001 | Noel et al. 2003 |
| AY335065 | Puerto Rico | 2001 | Noel et al. 2003 |
| AY335068 | Puerto Rico | 2001 | Noel et al. 2003 |
| AY335069 | Puerto Rico | 2001 | Noel et al. 2003 |
| AY335070 | Puerto Rico | 2001 | Noel et al. 2003 |
| AY335072 | Puerto Rico | 2001 | Noel et al. 2003 |
| AY335073 | Puerto Rico | 2001 | Noel et al. 2003 |
| AY335074 | Puerto Rico | 2001 | Noel et al. 2003 |
| AY335075 | Puerto Rico | 2001 | Noel et al. 2003 |
| AY335076 | Puerto Rico | 2001 | Noel et al. 2003 |
| AY335077 | Puerto Rico | 2001 | Noel et al. 2003 |
| AY335078 | Puerto Rico | 2001 | Noel et al. 2003 |
| AY335079 | Puerto Rico | 2001 | Noel et al. 2003 |
| AY335080 | Puerto Rico | 2001 | Noel et al. 2003 |
| AY335081 | Puerto Rico | 2001 | Noel et al. 2003 |
| AY335085 | Puerto Rico | 2001 | Noel et al. 2003 |
| AY335087 | Puerto Rico | 2001 | Noel et al. 2003 |
| AY335088 | Puerto Rico | 2001 | Noel et al. 2003 |
| AY335089 | Puerto Rico | 2001 | Noel et al. 2003 |
| AY335090 | Puerto Rico | 2001 | Noel et al. 2003 |
| AY335091 | Puerto Rico | 2001 | Noel et al. 2003 |
| AY335092 | Puerto Rico | 2001 | Noel et al. 2003 |
| AY335093 | Puerto Rico | 2001 | Noel et al. 2003 |
| AY335094 | Puerto Rico | 2001 | Noel et al. 2003 |
| AY335097 | Puerto Rico | 2001 | Noel et al. 2003 |
| AY335098 | Puerto Rico | 2001 | Noel et al. 2003 |
| AY335101 | Puerto Rico | 2001 | Noel et al. 2003 |
| AY335102 | Puerto Rico | 2001 | Noel et al. 2003 |
| AY335103 | Puerto Rico | 2001 | Noel et al. 2003 |
| AY335104 | Puerto Rico | 2001 | Noel et al. 2003 |
| AY335105 | Puerto Rico | 2001 | Noel et al. 2003 |
| AY335106 | Puerto Rico | 2001 | Noel et al. 2003 |
| AY335107 | Puerto Rico | 2001 | Noel et al. 2003 |
| AY335108 | Puerto Rico | 2001 | Noel et al. 2003 |
| AY335110 | Puerto Rico | 2001 | Noel et al. 2003 |
| AY335111 | Puerto Rico | 2001 | Noel et al. 2003 |
| AY335112 | Puerto Rico | 2001 | Noel et al. 2003 |
| AY335113 | Puerto Rico | 2001 | Noel et al. 2003 |
| AY335114 | Puerto Rico | 2001 | Noel et al. 2003 |
| AY335115 | Puerto Rico | 2001 | Noel et al. 2003 |
| AY335119 | Puerto Rico | 2001 | Noel et al. 2003 |
| AY335120 | Puerto Rico | 2001 | Noel et al. 2003 |
| AY335121 | Puerto Rico | 2001 | Noel et al. 2003 |
| AY335122 | Puerto Rico | 2001 | Noel et al. 2003 |
| AY335123 | Puerto Rico | 2001 | Noel et al. 2003 |
| AY335124 | Puerto Rico | 2001 | Noel et al. 2003 |
| AY335125 | Puerto Rico | 2001 | Noel et al. 2003 |
| AY335126 | Puerto Rico | 2001 | Noel et al. 2003 |
| AY335127 | Puerto Rico | 2001 | Noel et al. 2003 |
| AY335128 | Puerto Rico | 2001 | Noel et al. 2003 |
| AY335130 | Puerto Rico | 2001 | Noel et al. 2003 |
| AY335131 | Puerto Rico | 2001 | Noel et al. 2003 |
| AY335133 | Puerto Rico | 2001 | Noel et al. 2003 |
| AY335134 | Puerto Rico | 2001 | Noel et al. 2003 |
| AY335135 | Puerto Rico | 2001 | Noel et al. 2003 |
| AY335137 | Puerto Rico | 2001 | Noel et al. 2003 |
| AY335138 | Puerto Rico | 2001 | Noel et al. 2003 |
| AY335139 | Puerto Rico | 2001 | Noel et al. 2003 |
| AY335141 | Puerto Rico | 2001 | Noel et al. 2003 |
| AY335143 | Puerto Rico | 2001 | Noel et al. 2003 |
| AY335144 | Puerto Rico | 2001 | Noel et al. 2003 |
| AY335145 | Puerto Rico | 2001 | Noel et al. 2003 |
| AY335146 | Puerto Rico | 2001 | Noel et al. 2003 |
| AY335147 | Puerto Rico | 2001 | Noel et al. 2003 |
| AY335148 | Puerto Rico | 2001 | Noel et al. 2003 |
| AY335149 | Puerto Rico | 2001 | Noel et al. 2003 |
| AY335150 | Puerto Rico | 2001 | Noel et al. 2003 |
| AY335151 | Puerto Rico | 2001 | Noel et al. 2003 |
| AY335152 | Puerto Rico | 2001 | Noel et al. 2003 |
| AY335153 | Puerto Rico | 2001 | Noel et al. 2003 |
| AY335154 | Puerto Rico | 2001 | Noel et al. 2003 |
| AY335155 | Puerto Rico | 2001 | Noel et al. 2003 |
| AY335156 | Puerto Rico | 2001 | Noel et al. 2003 |
| AY335157 | Puerto Rico | 2001 | Noel et al. 2003 |
| AY755462/AY755492 | Puerto Rico | 2001 | Tirado et al. 2005 |
| AY755463/AY755493 | Puerto Rico | 2001 | Tirado et al. 2005 |
| AY755464/AY755494 | Puerto Rico | 2001 | Tirado et al. 2005 |
| AY755465/AY755495 | Puerto Rico | 2001 | Tirado et al. 2005 |
| AY755466/AY755496 | Puerto Rico | 2001 | Tirado et al. 2005 |
| AY755467/AY755497 | Puerto Rico | 2001 | Tirado et al. 2005 |
| AY755468/AY755498 | Puerto Rico | 2001 | Tirado et al. 2005 |
| AY755469/AY755499 | Puerto Rico | 2001 | Tirado et al. 2005 |
| AY755470/AY755500 | Puerto Rico | 2001 | Tirado et al. 2005 |
| AY755471/AY755501 | Puerto Rico | 2001 | Tirado et al. 2005 |
| AY755472/AY755482 | Puerto Rico | 2001 | Tirado et al. 2005 |
| AY755473/AY755483 | Puerto Rico | 2001 | Tirado et al. 2005 |
| AY755474/AY755484 | Puerto Rico | 2001 | Tirado et al. 2005 |
| AY755475/AY755485 | Puerto Rico | 2001 | Tirado et al. 2005 |
| AY755476/AY755486 | Puerto Rico | 2001 | Tirado et al. 2005 |
| AY755477/AY755487 | Puerto Rico | 2001 | Tirado et al. 2005 |
| AY755478/AY755488 | Puerto Rico | 2001 | Tirado et al. 2005 |
| AY755479/AY755489 | Puerto Rico | 2001 | Tirado et al. 2005 |
| AY755480/AY755490 | Puerto Rico | 2001 | Tirado et al. 2005 |
| AY755481/AY755491 | Puerto Rico | 2001 | Tirado et al. 2005 |
| DQ832138 | Puerto Rico | 2005 | Viani et al. 2006 |
|  |  |  |  |
| AY267305 | St. Lucia | 2000 | Vaughan et al. 2003 |
| AY267306 | St. Lucia | 2000 | Vaughan et al. 2003 |
| AY267322 | St. Lucia | 2000 | Vaughan et al. 2003 |
| AY267329 | St. Lucia | 2000 | Vaughan et al. 2003 |
|  |  |  |  |
| AY267315 | St. Vincent | 2000 | Vaughan et al. 2003 |
| AY267316 | St. Vincent | 2000 | Vaughan et al. 2003 |
| AY267317 | St. Vincent | 2000 | Vaughan et al. 2003 |
| AY267318 | St. Vincent | 2000 | Vaughan et al. 2003 |
|  |  |  |  |
| AY267259 | Trinidad & Tobago | 2000 | Vaughan et al. 2003 |
| AY267260 | Trinidad & Tobago | 2000 | Vaughan et al. 2003 |
| AY267261 | Trinidad & Tobago | 2000 | Vaughan et al. 2003 |
| AY267262 | Trinidad & Tobago | 2000 | Vaughan et al. 2003 |
| AY267263 | Trinidad & Tobago | 2000 | Vaughan et al. 2003 |
| AY267264 | Trinidad & Tobago | 2000 | Vaughan et al. 2003 |
| AY267266 | Trinidad & Tobago | 2000 | Vaughan et al. 2003 |
| AY267267 | Trinidad & Tobago | 2000 | Vaughan et al. 2003 |
| AY267269 | Trinidad & Tobago | 2000 | Vaughan et al. 2003 |
| AY267270 | Trinidad & Tobago | 2000 | Vaughan et al. 2003 |
| AY267271 | Trinidad & Tobago | 2000 | Vaughan et al. 2003 |
| AY267272 | Trinidad & Tobago | 2000 | Vaughan et al. 2003 |
| AY267273 | Trinidad & Tobago | 2000 | Vaughan et al. 2003 |
| AY267274 | Trinidad & Tobago | 2000 | Vaughan et al. 2003 |
| AY267275 | Trinidad & Tobago | 2000 | Vaughan et al. 2003 |
| AY267276 | Trinidad & Tobago | 2000 | Vaughan et al. 2003 |
| AY267277 | Trinidad & Tobago | 2000 | Vaughan et al. 2003 |
| AY267278 | Trinidad & Tobago | 2000 | Vaughan et al. 2003 |
| AY267279 | Trinidad Tobago | 2000 | Vaughan et al. 2003 |
| AY267280 | Trinidad Tobago | 2000 | Vaughan et al. 2003 |
| AY267281 | Trinidad Tobago | 2000 | Vaughan et al. 2003 |
| AY267282 | Trinidad Tobago | 2000 | Vaughan et al. 2003 |
| AY267283 | Trinidad Tobago | 2000 | Vaughan et al. 2003 |
| AY267285 | Trinidad Tobago | 2000 | Vaughan et al. 2003 |
| AY267287 | Trinidad Tobago | 2000 | Vaughan et al. 2003 |
| AY267288 | Trinidad Tobago | 2000 | Vaughan et al. 2003 |
| AY267289 | Trinidad Tobago | 2000 | Vaughan et al. 2003 |
| AY267290 | Trinidad Tobago | 2000 | Vaughan et al. 2003 |
| AY267291 | Trinidad Tobago | 2000 | Vaughan et al. 2003 |
| AY267292 | Trinidad Tobago | 2000 | Vaughan et al. 2003 |
| AY267293 | Trinidad Tobago | 2000 | Vaughan et al. 2003 |
| AY267301 | Trinidad Tobago | 2000 | Vaughan et al. 2003 |
| AY267302 | Trinidad Tobago | 2000 | Vaughan et al. 2003 |
| EU439730 | Trinidad Tobago | 2000 | Vaughan et al. 2003 |
| EU439731 | Trinidad Tobago | 2000 | Vaughan et al. 2003 |
| EU439732 | Trinidad Tobago | 2000 | Vaughan et al. 2003 |
| EU439733 | Trinidad Tobago | 2000 | Vaughan et al. 2003 |
| EU439734 | Trinidad Tobago | 2000 | Vaughan et al. 2003 |
| EU439735 | Trinidad Tobago | 2000 | Vaughan et al. 2003 |
| EU439736 | Trinidad Tobago | 2000 | Vaughan et al. 2003 |
| EU439737 | Trinidad Tobago | 2000 | Vaughan et al. 2003 |
| EU439738 | Trinidad Tobago | 2003 | Vaughan et al. 2003 |
| EU439739 | Trinidad Tobago | 2003 | Vaughan et al. 2003 |
| EU439740 | Trinidad Tobago | 2000 | Vaughan et al. 2003 |
| EU439741 | Trinidad Tobago | 2000 | Vaughan et al. 2003 |
| EU439742 | Trinidad Tobago | 2000 | Vaughan et al. 2003 |
| EU439743 | Trinidad Tobago | 2000 | Vaughan et al. 2003 |
| EU439744 | Trinidad Tobago | 2000 | Vaughan et al. 2003 |
| EU439745 | Trinidad Tobago | 2000 | Vaughan et al. 2003 |
| EU439746 | Trinidad Tobago | 2000 | Vaughan et al. 2003 |
| EU439747 | Trinidad Tobago | 2001 | Vaughan et al. 2003 |
| EU439748 | Trinidad Tobago | 2001 | Vaughan et al. 2003 |
| EU439749 | Trinidad Tobago | 2001 | Vaughan et al. 2003 |
| EU439750 | Trinidad Tobago | 2000 | Vaughan et al. 2003 |
| EU439751 | Trinidad Tobago | 2000 | Vaughan et al. 2003 |
| EU439752 | Trinidad Tobago | 2000 | Vaughan et al. 2003 |
| EU439753 | Trinidad Tobago | 2000 | Vaughan et al. 2003 |
| EU439754 | Trinidad Tobago | 2000 | Vaughan et al. 2003 |
| EU439755 | Trinidad Tobago | 2000 | Vaughan et al. 2003 |
| EU439756 | Trinidad Tobago | 2000 | Vaughan et al. 2003 |
| EU439757 | Trinidad Tobago | 2000 | Vaughan et al. 2003 |
| EU439758 | Trinidad Tobago | 2000 | Vaughan et al. 2003 |
| EU439759 | Trinidad Tobago | 2000 | Vaughan et al. 2003 |
| EU839606 | Trinidad Tobago | 2000 | Vaughan et al. 2003 |
| EU839607 | Trinidad Tobago | 2000 | Vaughan et al. 2003 |
| EU839608 | Trinidad Tobago | 2001 | Vaughan et al. 2003 |
| EU839609 | Trinidad Tobago | 2000 | Vaughan et al. 2003 |
| EU839610 | Trinidad Tobago | 2001 | Vaughan et al. 2003 |

**REFERENCES**

1. Ahumada-Ruiz S, Flores-Figueroa D, Toala-González I, Thomson MM (2009) Analysis of HIV-1 *pol* sequences from Panama: Identification of phylogenetic clusters within subtype B and detection of antiretroviral drug resistance mutations. Infect Genet Evol 9:933-94.
2. Castro E, Lorenzana I, Moreno M, Echeverria G (2005) Highly conserved HIV-1 subtype B strains circulating in Honduras. Poster Exhibition: The 3rd IAS Conference on HIV Pathogenesis and Treatment: Abstract no. MoPe14.1B01
3. Cuevas MT, Ruibal I, Villahermosa ML, Diaz H, Delgado E, Vazquez-de Parga E, Perez-Alvarez L, Blanco de Armas M, Cuevas L, Medrano L, Noa E, Osmanov S, Najera R, Thompson MM (2002) High HIV-1 genetic diversity in Cuba. AIDS 16:1643-1653.
4. Eyzaguirre L, Brouwer KC, Nadai Y, Patterson TL, Ramos R, Firestone Cruz M, Orozovich P, Strathdee SA, Carr JK (2007) First molecular surveillance report of HIV type 1 in injecting drug users and female sex workers along the U.S.-Mexico border. AIDS Res Hum Retroviruses 23:331-334.
5. Gittens MV, Roth WW, Roach T, Stringer HG Jr, Pieniazek D, Bond, Levett PN (2003) The molecular epidemiology and drug resistance determination of HIV type 1 subtype B infection in Barbados. AIDS Res Hum Retroviruses. 19:313-319.
6. Gomez-Carrillo M, Pampuro S, Duran A, Losso M, Harris DR, Read JS, Duarte G, De Souza R, Soto-Ramirez L, Salomon H (2006) Analysis of HIV type 1 diversity in pregnant women from four Latin American and Caribbean countries. AIDS Res Hum Retroviruses 22:1186-1191.
7. Hamilton CL, Eyzaguirre LM, Amarakoon II, Figueroa P, Duncan J, Carr JK, Roye ME (2012) Analysis of protease and reverse transcriptase genes of HIV for antiretroviral drug resistance in Jamaican adults. AIDS Res Hum Retroviruses. 28:923-927.
8. Holguín A, Erazo K, Escobar G, de Mulder M, Yebra G, Martín L, Jovel LE, Castaneda L, Pérez E; Proyecto Esther (2011) Drug resistance prevalence in human immunodeficiency virus type 1 infected pediatric populations in Honduras and El Salvador during 1989-2009. Pediatr Infect Dis J 30:82-87.
9. Lloyd B, O’Connell RJ, Michael NL, Aviles R, Palou E, Hernandez R, Cooley J, Jagodzinski LL (2008) Prevalence of resistance mutations in HIV-1-infected Hondurans at the beginning of the National Antiretroviral Therapy Program. AIDS Res Hum Retroviruses 24: 529-535.
10. Machado LY, Blanco M, Dubed M, Díaz HM, Ruiz NM, Váldes N, Romay D, Lobaina LI (2011) HIV type 1 genetic diversity in newly diagnosed Cuban patients. AIDS Res Hum Retroviruses 28:956-960.
11. Machado DM, Delwart EL, Diaz RS, de Oliveira CF, Alves K, Rawal BD, Sullivan M, Gwinn M, Clark KA, Busch MP (2002) Use of the sensitive/less-sensitive (detuned) EIA strategy for targeting genetic analysis of HIV-1 to recently infected blood donors. AIDS 16:113-119.
12. Murillo W, de Rivera IL, Parham L, Jovel E, Palou E, Karlsson AC, Albert J (2010) Prevalence of drug resistance and importance of viral load measurements in Honduran HIV-infected patients failing antiretroviral treatment. HIV Medicine 11:95-103.
13. Nadai Y, Eyzaguirre LM, Sill A, Cleghorn F, Nolte C, Charurat M, Collado-Chastel S, Jack N, Bartholomew C,Pape JW, Figueroa P, Blattner WA, Carr JK (2009) HIV-1 Epidemic in the Caribbean is dominated by subtype B. PLoS ONE 4: e4814.
14. Noel RJ Jr, Chaudhary S, Rodriguez N, Kumar A, Yamamura Y (2003) Phylogenetic relationships between Puerto Rico and continental USA HIV-1 *pol* sequences: a shared HIV-1 infection. Cell Mol Biol 49:1193-1198.
15. Parham L, de Rivera IL, Murillo W, Naver L, Largaespada N, Albert J, Karlsson AC (2011) High relevance of drug resistance in HIV type 1-infected children born in Honduras and Belize 2001 to 2004. AIDS Res Hum Retroviruses 27:1055-1059.
16. Perez L, Thompson MM, Bleda MJ, Aragones C, Gonzalez Z, Perez J, Sierra M, Casado G, Delgado E, Najera R (2006) HIV type 1 molecular epidemiology in Cuba: High genetic diversity, frequent mosaicism, and recent expansion of BG intersubtype recombinant forms. AIDS Res Hum Retroviruses 22:724-733.
17. Roye ME, Amarakoon II, Hamilton CL, Eyzaguirre LM, Figueroa P, Carr JK (2011) Genotypic characterization of HIV type 1 in Jamaica. AIDS Res Hum Retroviruses 27:91-95.
18. Tirado G, Jove G, Reyes E, Sepulveda G, Yamamura Y, Singh DP, Kumar A (2005) Differential evolution of cell-associated virus in blood and genital tract of HIV-infected females undergoing HAART. Virology 334:299-305.
19. Valle-Bahena OM, Ramos-Jiménez J, Ortiz-López R, Revol A, Lugo-Trampe A, Barrera-Saldaña HA, Rojas-Martínez A (2006) Frequency of protease and reverse transcriptase drug resistance mutations in naïve HIV-infected patients. Arch Med Res 37:1022-1027.
20. Vaughan HE, Cane P, Pillay D, Tedder RS (2003) Characterization of HIV Type 1 Clades in the Caribbean Using pol Gene Sequences. AIDS Res Hum Retroviruses 19:929-932.
21. Viani RM, Peralta L, Aldrovandi G, Kapogiannis BG, Mitchell R, Spector SA, Lie YS, Weidler JM, Bates MP, Liu N, Wilson CM, and the Adolescent Medicine Trials Network for HIV/AIDS Interventions (2006) Prevalence of primary HIV-1 drug resistance among recently infected adolescents: A multicentre Adolescent Medicine Trials Network for HIV/AIDS Interventions study. 194:1505-1509.
