# Supplementary material for: Reconstructing the Timing and Dispersion Routes of HIV-1 Subtype B Epidemics in The Caribbean and Central America: A Phylogenetic Story
Source: PLoS One. 2013 Jul 9;8(7):e69218. doi: 10.1371/journal.pone.0069218 (PMC3706403; doi:10.1371/journal.pone.0069218)
Supplement: Table S2 — Nucleotide substitution rate, TMRCA and HIV-1B epidemic geographical origin estimates for the ten “balanced” replicates of the polp data sets from Central American (n = 69), and Caribbean (n = 155) countries. (DOCX) [file pone.0069218.s003.docx]

**Table S2.** Nucleotide substitution rate, TMRCA and HIV-1B epidemic geographical origin estimates for the ten “balanced” replicates of the *pol_p_* data sets from Central American (n=69), and Caribbean (n=155) countries.

|  | **Replicate** | **Substitution rate^a^** | **MRCA^b^** | **Estimated Origin^c^** |
| --- | --- | --- | --- | --- |
| **Central America** | **1** | 3.10x10^-3^ (1.95-4.27x10^-3^) | 1988 (1981-1994) | Honduras |
|  | **2** | 3.26x10^-3^ (2.12-4.44x10^-3^) | 1989 (1983-1994) | Honduras |
|  | **3** | 2.08x10^-3^ (2.00-4.15x10^-3^) | 1986 (1962-1992) | Honduras |
|  | **4** | 3.16x10^-3^ (1.86-4.53x10^-3^) | 1986 (1979-1994) | El Salvador |
|  | **5** | 3.12x10^-3^ (1.72-4.52x10^-3^) | 1987 (1977-1994) | Honduras |
|  | **6** | 3.07x10^-3^ (1.68-4.43x10^-3^) | 1987 (1977-1994) | Honduras |
|  | **7** | 2.72x10^-3^ (1.84-3.69x10^-3^) | 1986 (1978-1992) | El Salvador |
|  | **8** | 2.33x10^-3^ (1.41-3.37x10^-3^) | 1983 (1973-1992) | Honduras |
|  | **9** | 2.60x10^-3^ (1.69-3.54x10^-3^) | 1983 (1976-1991) | El Salvador |
|  | **10** | 2.39x10^-3^ (1.07-3.70x10^-3^) | 1985 (1963-1993) | Honduras |
|  |  |  |  |  |
| **The Caribbean** | **1** | 1.69x10^-3^ (1.13-2.33x10^-3^) | 1966 (1963-1971) | Puerto Rico, Antigua |
|  | **2** | 1.39x10^-3^ (0.92-1.77x10^-3^) | 1942 (1939-1973) | Puerto Rico, Antigua |
|  | **3** | 2.58x10^-3^ (1.49-3.50x10^-3^) | 1974 (1968-1980) | Puerto Rico, Antigua |
|  | **4** | 1.26x10^-3^ (0.75-1.82x10^-3^) | 1941 (1924-1978) | Puerto Rico, Antigua |
|  | **5** | 2.39x10^-3^ (1.52-3.06x10^-3^) | 1970 (1961-1982) | Puerto Rico, Antigua |
|  | **6** | 1.73x10^-3^ (1.31-2.21x10^-3^) | 1963 (1954-1975) | Puerto Rico, Antigua |
|  | **7** | 1.60x10^-3^ (1.20-2.13x10^-3^) | 1962 (1948-1970) | Puerto Rico, Antigua |
|  | **8** | 2.17x10^-3^ (1.64-2.78x10^-3^) | 1971 (1964-1980) | Puerto Rico, Antigua |
|  | **9** | 1.37x10^-3^ (0.92-1.90x10^-3^) | 1951 (1937-1960) | Haiti, Antigua |
|  | **10** | 1.85x10^-3^ (1.33-2.68x10^-3^) | 1963 (1957-1980) | Haiti, Antigua |

^a^ Nucleotide substitution rate (substitutions per site per year), with 95% HPD in parenthesis.

^b^ Most recent common ancestor (date), with 95% HPD in parenthesis.

^c^ Origin of the HIV-1B epidemic based on the phylogeographic reconstructions.
